# Rice carotenoid biofortification and yield improvement conferred by endosperm-specific overexpression of *OsGLK1*

**DOI:** 10.3389/fpls.2022.951605

**Published:** 2022-07-15

**Authors:** Zhenjun Li, Jianjie Gao, Bo Wang, Jing Xu, Xiaoyan Fu, Hongjuan Han, Lijuan Wang, Wenhui Zhang, Yongdong Deng, Yu Wang, Zehao Gong, Yongsheng Tian, Rihe Peng, Quanhong Yao

**Affiliations:** Shanghai Key Laboratory of Agricultural Genetics and Breeding, Agro-Biotechnology Research Institute, Shanghai Academy of Agricultural Sciences, Shanghai, China

**Keywords:** grain yield, *OsGLK1*, plastids, rice endosperm, carotenoids

## Abstract

Carotenoids, indispensable isoprenoid phytonutrients, are synthesized in plastids and are known to be deficient in rice endosperm. Many studies, involving transgenic manipulations of carotenoid biosynthetic genes, have been performed to obtain carotenoid-enriched rice grains. Nuclear-encoded GOLDEN2-LIKE (GLK) transcription factors play important roles in the regulation of plastid and thylakoid grana development. Here, we show that endosperm-specific overexpression of rice *GLK1* gene (*OsGLK1*) leads to enhanced carotenoid production, increased grain yield, but deteriorated grain quality in rice. Subsequently, we performed the bioengineering of carotenoids biosynthesis in rice endosperm by introducing other three carotenogenic genes, *tHMG1*, *ZmPSY1*, and *PaCrtI*, which encode the enzymes truncated 3-hydroxy-3-methylglutaryl-CoA reductase, phytoene synthase, and phytoene desaturase, respectively. Transgenic overexpression of all four genes (*OsGLK1*, *tHMG1*, *ZmPSY1*, and *PaCrtI*) driven by rice endosperm-specific promoter *GluB-1* established a mini carotenoid biosynthetic pathway in the endosperm and exerted a roughly multiplicative effect on the carotenoid accumulation as compared with the overexpression of only three genes (*tHMG1*, *ZmPSY1*, and *PaCrtI*). In addition, the yield enhancement and quality reduction traits were also present in the transgenic rice overexpressing the selected four genes. Our results revealed that *OsGLK1* confers favorable characters in rice endosperm and could help to refine strategies for the carotenoid and other plastid-synthesized micronutrient fortification in bioengineered plants.

## Introduction

Carotenoids are a diverse group of hydrophobic isoprenoid pigments ubiquitously synthesized by plants, algae, fungi, and bacteria (Sun et al., 2018). Carotenoids play critical roles in light-harvesting and protection of the photosynthetic apparatus against damage by high light and/or temperature stress (Nisar et al., 2015). Carotenoids provide precursors for the biosynthesis of plant hormones and serve as important contributors to visual appeal of ornamental plants and nutritional qualities of fruits and vegetables (Cazzonelli and Pogson, 2010; Ruiz-Sola and Rodríguez-Concepción, 2012).

Most cereal crops contain only trace levels of dietary carotenoids. Carotenoids are currently under intense scrutiny regarding their potential to reduce the risks of some chronic diseases and prevent retinol (vitamin A) deficiency. Thus, a great deal of research effort has been made to modify the types and levels of carotenoids in economically important crops (Paine et al., 2005). The carotenoid biosynthesis at the molecular level in higher plants has been well elucidated. Plant carotenoids are isopentenyl diphosphate (IPP)-derived molecules, and two pathways independently contribute to the production of IPP in plants: the cytosolic mevalonic acid (MVA) pathway and the plastidic methylerythritol 4-phosphate (MEP) pathway (Hemmerlin et al., 2012). In plant cells, plastidic MEP pathway is the major pathway for carotenoid biosynthesis and storage (Lopez-Juez and Pyke, 2005). Many studies have been focused on increasing metabolic flux in plastids by overexpression of one or more crucial enzymes within MEP pathway for carotenoid accumulation (Shewmaker et al., 1999; Zhai et al., 2016; Paul et al., 2017). However, very few genes that are not from the two pathways have been well characterized for their influence on carotenoid production in plants.

The endosperm is the largest component of the whole cereal grains, and serves as major food staple, but it is deficient in many nutritionally valuable biochemical compounds such as vitamins, minerals, and carotenoids (Zhu et al., 2007, 2008). Previous studies have shown that some carotenoids are not essential but beneficial to health, whereas some, such as pro-vitamin A carotenoids, have been considered as essential nutrients because they are unable to be synthesized *de novo* by humans and must be acquired through the diet (Fraser and Bramley, 2004; Bai et al., 2011). Rice (*Oryza sativa L.*) is a major cereal food crop in most of the developing countries. Due to the absence of carotenoids in rice endosperm, the consumption of rice as an integral part of diet is often accompanied by vitamin A deficiency (Underwood and Arthur, 1996; Farré et al., 2010).

Biofortification is an economically effective process by which crops are bred in a way that increases the production of micronutrients or phytonutrients *via* genetic engineering (Bouis and Saltzman, 2017). Biofortified crops enriched with phytonutrients, such as flavonoid-enriched tomato (Le Gall et al., 2003), astaxanthin-enriched rice (Zhu et al., 2018), vitamin B_6_-enriched rice (Mangel et al., 2019), and anthocyanin-enriched wheat (Sharma et al., 2020), may have the potential to bring great benefits to human health (Hirschi, 2009; De Steur et al., 2015; Fang et al., 2018).

The availability of a large number of carotenogenic genes makes it possible to modify and engineer the carotenoid biosynthetic pathways in host plants. Combinatorial coexpression by a single expression vector harboring all the target genes is commonly regarded as a time-saving and highly efficient plant transformation strategy in comparison with cotransformation of multiple constructs and transgene stacking by crossing (Fang et al., 2018).

The overexpression of carotenoid biosynthetic genes can boost carotenoid accumulation in staple crops if upstream pathways could supply sufficient isoprenoid precursors (Rodríguez-Concepción, 2010). Downstream contributors of carotenoid metabolism and sequestration serve to deplete the carotenoid pool and drive the reaction forward (Auldridge et al., 2006; Campbell et al., 2010). The first committed step of carotenoid biosynthesis is initiated by the condensation of two geranylgeranyl diphosphate (GGPP) molecules. Phytoene synthase (PSY) is the rate-limiting enzyme that catalyzes this reaction in the carotenoid pathway (Cazzonelli and Pogson, 2010). There is also limited flux in the subsequent desaturation reaction, which eventually results in the production of lycopene (Nisar et al., 2015). In order to promote phytoene-to-lycopene conversion, the bacterial phytoene desaturase enzyme *CrtI* was coexpressed with *PSY* in rice endosperm and finally contributed to the accumulation of β-carotene (Paine et al., 2005). Although the expression of these two enzymes increases metabolic flux in the early part of the carotenoid biosynthesis pathway, the exhaustion of the precursor pool remains an unresolved problem (Ye et al., 2000; Paine et al., 2005; Schaub et al., 2005).

3-Hydroxy-3-methylglutaryl coenzyme A reductase (HMGR) governs the MVA pathway-derived isoprenoids and plays a crucial role in isoprenoid biosynthesis. Transgenic tomatoes overexpressing plant *HMGR* without also overexpressing *PSY* and *CrtI* genes show IPP accumulation and no carotenoid accumulation (Enfissi et al., 2005). Although *HMGR* localizes in the cytosol and belongs to the MVA pathway, the overexpression of *HMGR* could significantly boost the production of IPP which may ultimately contribute to the downstream carotenoid biosynthesis. To address this challenge, we heterologously expressed the maize (*Zea mays*) *PSY* (*ZmPSY1*) and *Pantoea ananatis CrtI* (*PaCrtI*) genes along with *tHMG1*, which encodes truncated HMGR (N-terminal 554-amino acid-deletion) from *Saccharomyces cerevisiae*, specifically in the endosperm to boost flux through the MVA pathway, which generates carotenoid precursors. It was found that the combined expression of *tHMG1*, *ZmPSY1*, and *PaCrtI* under the control of endosperm-specific promoter *GluB-1* significantly promote carotenoid accumulation in rice endosperm, confirming that the participation of *tHMG1* in supply of isoprenoid precursors created a metabolic sink for the sustainable production of carotenoids (Tian et al., 2019).

*De novo* carotenoids are synthesized within almost all types of plastids, most importantly, chloroplasts and chromoplasts, in leaves, fruits, flowers, roots, and seeds (Yuan et al., 2015; Sun et al., 2018). Plastid identity is a key carotenoid-defining parameter which plays essential roles in governing carotenoid stability and composition. In chloroplasts, the majority of carotenoids are localized in the thylakoid membranes and plastoglobules, which are identified as the sites of carotenoid storage (Chaudhary et al., 2009). The *GOLDEN2-LIKE* (*GLK*) genes encode transcription factors that regulate plastid development and chlorophyll levels in diverse species, namely in the monocot maize and in the eudicot *Arabidopsis*. Defects in plastid development and chlorophyll biosynthesis usually result in compromised carotenoid biosynthesis (Langdale and Kidner, 1994; Hall et al., 1998; Rossini et al., 2001; Fitter et al., 2002). Previous studies showed that overexpression of *GLK*s could significantly promote chlorophyll biosynthesis and increase chloroplast number and thylakoid grana stacks in plant (Fitter et al., 2002; Yasumura et al., 2005; Nguyen et al., 2014; Chen et al., 2016).

**Figure 1:**
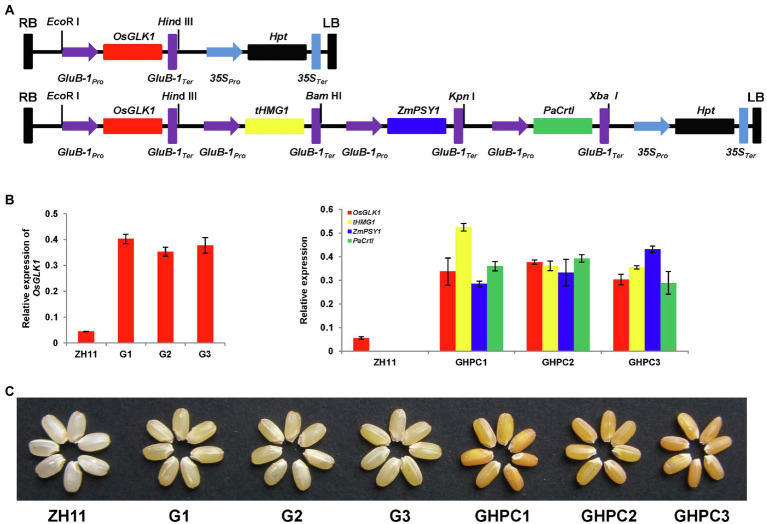
Engineering the biosynthesis of carotenoid in rice endosperm. **(A)** Schematic representation of the gene cassettes in the two plasmids used for rice transformation. Upper: vector harboring *OsGLK1*; Lower: vector harboring four genes (*OsGLK1*, *tHMG1*, *ZmPSY1*, and *PaCrtI*). Target genes were all driven by endosperm-specific promoter *GluB-1*. **(B)** Expression levels of the transgenes in homologous T3 lines of G and GHPC rice endosperms. RNA samples were prepared from endosperms of developing seeds (9 days after pollination) of the homologous T3 lines. The expression levels of the transgenes were normalized to the expression of reference gene *OsActin*. Values represent means of three replicates ± SD. **(C)** Representative phenotype of transgenic rice seeds showing altered color due to carotenoid accumulation. Two different endosperm phenotypes were observed. The light-yellow endosperm expressed *OsGLK1* (genotype G), and the orange-yellow endosperm coexpressed *OsGLK1*, *tHMG1*, *ZmPSY1*, and *PaCrtI* (genotype GHPC).

Therefore, we expressed the *OsGLK1* gene (genotype G) specifically in the rice endosperm to investigate its roles in carotenoid production. As a comparison, we also create another construct containing four genes encoding *OsGLK1*, *tHMG1*, *ZmPSY1*, and *PaCrtI* (genotype GHPC) to investigate their additive effects on the biofortification of carotenoids in rice endosperm (Figure 1A). It was found that *OsGLK1* significantly enhanced carotenoid production in rice endosperm and increased grain yield. However, the grain quality was adversely affected. Meanwhile, the combined overexpression of *OsGLK1*, *tHMG1*, *ZmPSY1*, and *PaCrtI* led to multiplicative effects on the carotenoid accumulation in rice endosperm compared with previous three-gene combination (*tHMG1*, *ZmPSY1*, and *PaCrtI*, genotype HPC) from our lab (Tian et al., 2019). Our data presented a conceptual and mechanistic basis to generate “New Golden Rice” and to update knowledge on the characterization of the carotenoid biosynthesis pathway.

## Materials and methods

### Vector construction

The transgenes, based on rice *GLK1* (GenBank: AAK50393), *S. cerevisiae* truncated *HMGR* (GenBank: AJS96703), maize *PSY1* (GenBank: AAB60314), and *P. ananatis CrtI* (GenBank: AHG94990) were chemically synthesized through PCR-based two-step DNA synthesis method (PTDS; Xiong et al., 2006). The codons of these genes were optimized in accordance with the codon usage frequencies of rice. Primers used for chemical synthesis of the four genes are listed in Supplementary Table S1. Then, the two gene expression cassettes (*G:OsGLK1* and *G:OsGLK1*-*G:tHMG1*-*G:ZmPSY1*-*G:PaCrtI*) were constructed by inserting the ORFs between an endosperm-specific promoter *GluB-1* and terminator using the PAGE-mediated overlap extension PCR method (Peng et al., 2014). The original expression vector for rice transformation was pCAMBIA1301 which was modified through the introduction of new multiple cloning sites by our lab to yield the binary vector pYP694 (Peng et al., 2014). Then, *pYP69-G:OsGLK1* (genotype G) was generated by inserting the *G:OsGLK1* expression cassette into pYP694 at the *Eco* RI and *Hin*d III sites. Then the *pYP69-G:tHMG1-G:ZmPSY1-G:PaCrtI* vector (genotype HPC) provided by Tian et al. (2019) was used as the donor vector for the introduction of *G:OsGLK1* to produce the four-gene construct *pYP69-G:OsGLK1-G:tHMG1-G:ZmPSY1-G:PaCrtI* (genotype GHPC). The detailed method was mentioned as previously described (Tian et al., 2019).

### Plant materials, strains, and chemicals

The rice conventional variety ZH11 was used for transformation of the two constructs described above. The *Agrobacterium tumefaciens* strain GV3101 was used for rice transformation. The field experiments were performed during the growing season in Shanghai and Hainan, China. Commercially available carotenoid standards, including α-carotene, β-carotene, lycopene, zeaxanthin, lutein, neoxanthin, and astaxanthin were purchased from Sigma Chemical Company (St. Louis, MO, United States).

### Carotenoid extraction and quantitative analysis by HPLC

Carotenoids were extracted from endosperms of different genotypes. The extraction method was described previously (Zhu et al., 2018). The HPLC analysis was performed using an Agilent 1,100 HPLC system (Agilent Technologies, CA, United States). The chromatographic separation was achieved with Agilent LC-C_18_ (4.6 mm × 250 mm, 5 μm) column and a gradient system with the mobile phase consisting of solvent A (acetonitrile) and solvent B (isopropanol) at a flow rate of 1 ml/min. The linear gradient was as follows: 0–10.0 min, 100% A; 10.0–13.0 min, 60% A; 13.0–20.0 min, 50% A; 20.0–30.0 min, 100% A. The UV spectra were recorded with a diode-array detector at 450 nm. Identification and peak assignment of each carotenoid component were primarily based on the comparison of their retention time and UV–visible spectrum data with that of standards.

### Metabolite profiling

Endosperms of mature ZH11 and G1 seeds were subjected to metabolomic analysis in our study. Metabolomic analysis of rice endosperms was performed using UHPLC-QTOFMS technology (Ultra-High-Performance Liquid Tandem Chromatography Quadrupole Time of Flight Mass Spectrometry). Three biological replicates were performed for each genotype, with each replicate being a pool of 5 g endosperms. The protocols for sample preparation have been described in detail (Wu et al., 2020; Liu et al., 2021). The UHPLC separation was carried out using a Waters ACQUITY UPLC HSS T3 column (100 × 2.1 mm, 1.8 μm). The AB Sciex QTOF mass spectrometer was used to acquire MS/MS spectra. Heatmaps and hierarchical cluster analysis were generated by Multi-Experiment Viewer (version 4.8.1).

### Transmission electron microscopy

The transmission electron microscopy observation was performed according to a protocol previously published (Luo et al., 2013) with a slight modification. Rice samples were first fixed overnight (4°C) in 2.5% (v/v) glutaraldehyde (pH 7.2), postfixed in 1% osmium tetroxide at 4°C for 1 h, followed by dehydration through an ethyl alcohol series. After dehydration, samples were embedded in epon araldite (resin) and polymerization was conducted at 40°C for 24 h. Samples were then transferred to fresh resin and hardened under nitrogen air at 60°C for 2 days, followed by sectioning of samples using Leica EM UC7 ultramicrotome (Wetzlar). Sections were stained with 5% uranyl acetate and imaged with a H7100FA transmission electron microscope (Hitachi).

### Scanning electron microscopy

Scanning electron microscopy observation was performed as described in the previous studies (Imai et al., 2006). Grain samples were transversely cut by a knife and coated with gold by E-100 ion sputter. Specimens were observed using a scanning electron microscope (SEM, VEGA3, TESCAN, Czech Republic). All procedures were conducted according to the manufacturer’s protocol.

### Expression analysis

Total RNA was extracted from 0.1 g of immature milky-stage endosperm (9 days after pollination) using an RNeasy Mini kit (Qiagen, Germany) according to the manufacturer’s instructions. All isolated RNA samples were treated with RNase-free DNase I (Promega, United States). Complementary DNA (cDNA) was synthesized from total RNA (2 μg) using a Quantitect reverse transcription kit (Qiagen) following the supplier’s protocol. The expression of the four transgenes (*OsGLK1*, *tHMG1*, *ZmPSY1*, and *PaCrtI*) and 11 endogenous carotenogenic genes (*OsPSY*, *OsPDS*, *OsZISO*, *OsZDS*, *OsCRTISO*, *OsLCYE*, *OsLCYB*, *OsBHY*, *OsEHY*, *OsZEP*, and *OsVED*) were investigated by qRT-PCR using SYBR Premix Ex Taq II (Takara, Japan) according to the supplier’s instructions. The expression of target genes were normalized to the reference gene *OsActin* (Li et al., 2019). PCR conditions for amplification of the four transgenes were as follows: 4 min at 95°C, followed by 35 cycles of 10 s at 95°C, 15 s at 59°C (54°C for the endogenous carotenogenic genes), 20 s at 72°C. Data are shown as means ± SD (three biological replicates). The information of primers used is shown in Supplementary Table S2. The loci of the 11 endogenous carotenogenic genes in The Rice Annotation Project Database^1^ were as follows: *OsPSY*, Os12g0626400; *OsPDS*, Os03g0184000; *OsZISO*, Os12g0405200; *OsZDS*, Os07g0204900; *OsCRTISO*, Os11g0572700; *OsLCYB*, Os02g0190600; *OsLCYE*, Os01g0581300; *OsBHY*, Os10g0533500; *OsEHY*, Os03g0125100; *OsZEP*, Os04g0448900; and *OsVDE*, Os04g0379700.

### Trait measurement

Fully filled grains were used for measuring the 1,000-grain weight. The grains with chalkiness were counted, and the percentage of chalky grains was calculated as the chalkiness percentage. For chalkiness degree, 50 grains with chalkiness were randomly selected, and the ratio of the chalkiness area to the whole kernel square for each grain was evaluated. In order to easily distinguish the chalkiness regions from other regions, the images of rice kernels were captured using a digital camera (Canon) and processed by Image J (version 1.8.0). The chalkiness degree was defined as the ratio of the chalkiness area to the whole kernel square. The graphical plots were prepared using GraphPad Prism software (Version 8.3.0).

## Results

### Generation of carotenoids in rice

*Agrobacterium*-mediated transformation of ZH11 was carried out to create transgenic plants. Nine independent G and 6 independent GHPC transgenic lines were obtained. Homozygous transgenic rice lines, harboring a single copy of targeted construct (Figure 1A), were selected for the subsequent studies. Subsequently, qRT-PCR analysis was performed to examine the transcript levels of the four transgenes in the endosperm of developing seeds. Since the transcript levels of all transgenes were greatly enhanced in the endosperm, we designated these lines as G1, G2, G3, GHPC1, GHPC2, and GHPC3 lines (Figure 1B). All transgenic rice produced light-yellow and orange-yellow endosperms for G and GHPC lines, respectively (Figure 1C).

### Composition of carotenoids and their contents in transgenic lines

Carotenoid concentrations in rice endosperms were measured by HPLC to ascertain the effect of *OsGLK1* on pigment accumulation. Seven major carotenoids (lycopene, α-carotene, β-carotene, lutein, zeaxanthin, neoxanthin, and astaxanthin) were quantified. Quantitative analysis of the contents of these components showed that overexpression of *OsGLK1* could significantly increase carotenoid production in rice endosperm. The total carotenoid content was 2.13, 2.37, and 2.32 μg/g dry weight (DW) in lines G1, G2, and G3, respectively. It needs to be noticed that the content of β-carotene increased to a greater extent in all G lines compared with the production of other carotenoid components (Table 1; Supplementary Figure S1). Metabolite profiling of rice endosperms of line G1 and ZH11 identified 89 metabolites with altered levels, only a small number of which showed changes greater than 2 folds, suggesting that critical metabolic activities were unaffected in *OsGLK1* overexpression plants (Supplementary Table S3; Supplementary Figure S2).

**Table 1:** Carotenoid content and composition of mature rice endosperms from different genotypes.

Composition (μg/g DW)	Genotype						
	ZH11	G1	G2	G3	GHPC1	GHPC2	GHPC3
α-Carotene	0 ± 0	0.247 ± 0.057^***^	0.237 ± 0.044^***^	0.243 ± 0.048^***^	9.472 ± 0.112^***^	9.547 ± 0.042^***^	8.839 ± 0.41^***^
β-Carotene	0.009 ± 0.001	1.474 ± 0.11^***^	1.48 ± 0.086^***^	1.572 ± 0.016^***^	28.158 ± 0.283^***^	28.774 ± 0.52^***^	26.227 ± 0.634^***^
Lycopene	0 ± 0	0.105 ± 0.009^***^	0.222 ± 0.012^***^	0.209 ± 0.018^***^	0.796 ± 0.001^***^	0.798 ± 0.002^***^	0.795 ± 0.001^***^
Zeaxanthin	0.005 ± 0.001	0.072 ± 0.003^***^	0.093 ± 0.006^***^	0.072 ± 0.007^***^	0.295 ± 0.003^***^	0.299 ± 0.016^***^	0.276 ± 0.013^***^
Lutein	0.025 ± 0.002	0.231 ± 0.01^***^	0.342 ± 0.007^***^	0.228 ± 0.006^***^	0.635 ± 0.004^***^	0.64 ± 0.023^***^	0.673 ± 0.027^***^
Total carotenoid	0.039 ± 0.004	2.128 ± 0.089^***^	2.373 ± 0.086^***^	2.323 ± 0.022^***^	39.356 ± 0.693^***^	40.058 ± 0.628^***^	36.81 ± 0.538^***^

Data are presented as means ± SD of three biological replicates. *p*-values produced by two-tailed Student’s *t*-test. DW, dry weight.

*** *p* < 0.001.

**Figure 2:**
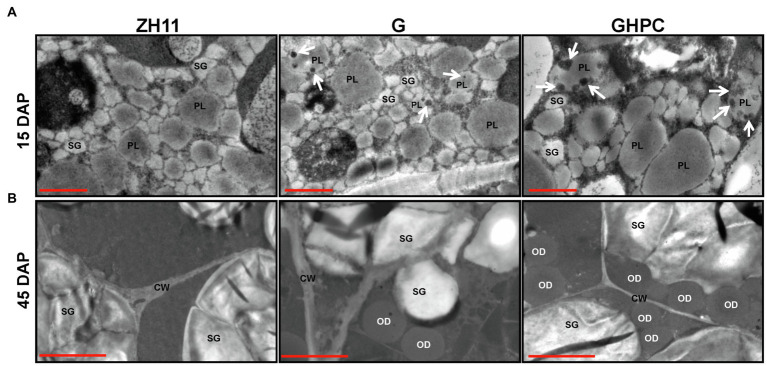
Transmission electron micrographs of rice endosperm. **(A)** The endosperm cells (15 DAP) of ZH11, G, and GHPC genotypes are shown. Scale bar = 1 μm. **(B)** The endosperm cells (45 DAP) of ZH11, G, and GHPC genotypes are shown. Scale bar = 2 μm. DAP, days after pollination. White arrows indicate the electron-dense plastoglobuli in plastids (PL). SG, starch grain; OD, oil droplet; CW, cell walls.

The GHPC lines produced the highest levels of carotenoids (39.36, 40.06, and 36.81 μg/g DW for lines GHPC1, GHPC2, and GHPC3, respectively; Table 1; Supplementary Figure S1). This revealed an increase in total carotenoids of nearly 3-fold compared to the previously reported HPC rice endosperms (up to 14.45 μg/g DW) from our lab (Tian et al., 2019), supporting the notion that introduction of the *OsGLK1* gene has a big impact on carotenoids production in rice. The total carotenoid contents ranged from 2.13 to 2.37 μg/g DW and 13.71–14.45 μg/g DW in G and HPC lines, respectively. However, the GHPC lines produced more than 40 μg/g DW of total carotenoids. These results indicate that *OsGLK1* might have roughly multiplicative, rather than additive effects on the carotenoids accumulation in our study. In addition, qRT-PCR analysis was performed to investigate the expression levels of *tHMG1*, *ZmPSY1*, and *PaCrtI* in HPC lines. The results showed that the transcript levels of the three genes in GHPC lines are comparable with those in HPC lines (Figure 1B; Supplementary Figure S3), which further justified the extraordinary effect of *OsGLK1* on carotenoid accumulation. Transmission electron microscopy revealed that a few electron-dense plastoglobuli (which may contain carotenoids) were visible inside some plastids of 15 DAP (days after pollination) endosperm in the genotypes G and GHPC. However, we can barely observe plastoglobuli in the ZH11 and HPC endosperms (Figure 2A; Supplementary Figure S4A). The formation of plastoglobuli in the plastids of transgenic rice endosperm might be, at least in part, due to the overexpression of *OsGLK1* in our study. In addition, TEM of G, HPC, and GHPC grains revealed numerous oil droplets (also called spherosomes) in the 45 DAP endosperm, while no oil droplets could be observed in ZH11 endosperm (Figure 2B; Supplementary Figure S4B). It was previously reported that the elevation of the predominant carotenoid is consistent with elevated plastid numbers (Nguyen et al., 2014). The multiplicative effect on the burst production of carotenoids imposed by *OsGLK1* might be due to the improved thylakoid membrane biogenesis and plastid structure and number. *GluB-1* promoter is commonly used for foreign gene expression only in the endosperm area (Washida et al., 1999). TEM was also employed to monitor the chloroplast development in 15 DAP seed coats of each genotype. As expected, no significant differences among ZH11, G and GHPC genotypes regarding the seed coat color were detected (Supplementary Figure S5A). The chloroplasts in seed coats from each genotype developed normally with no obvious phenotypic difference (Supplementary Figures S5B,C).

### Expression of endogenous carotenoid biosynthetic genes

**Figure 3:**
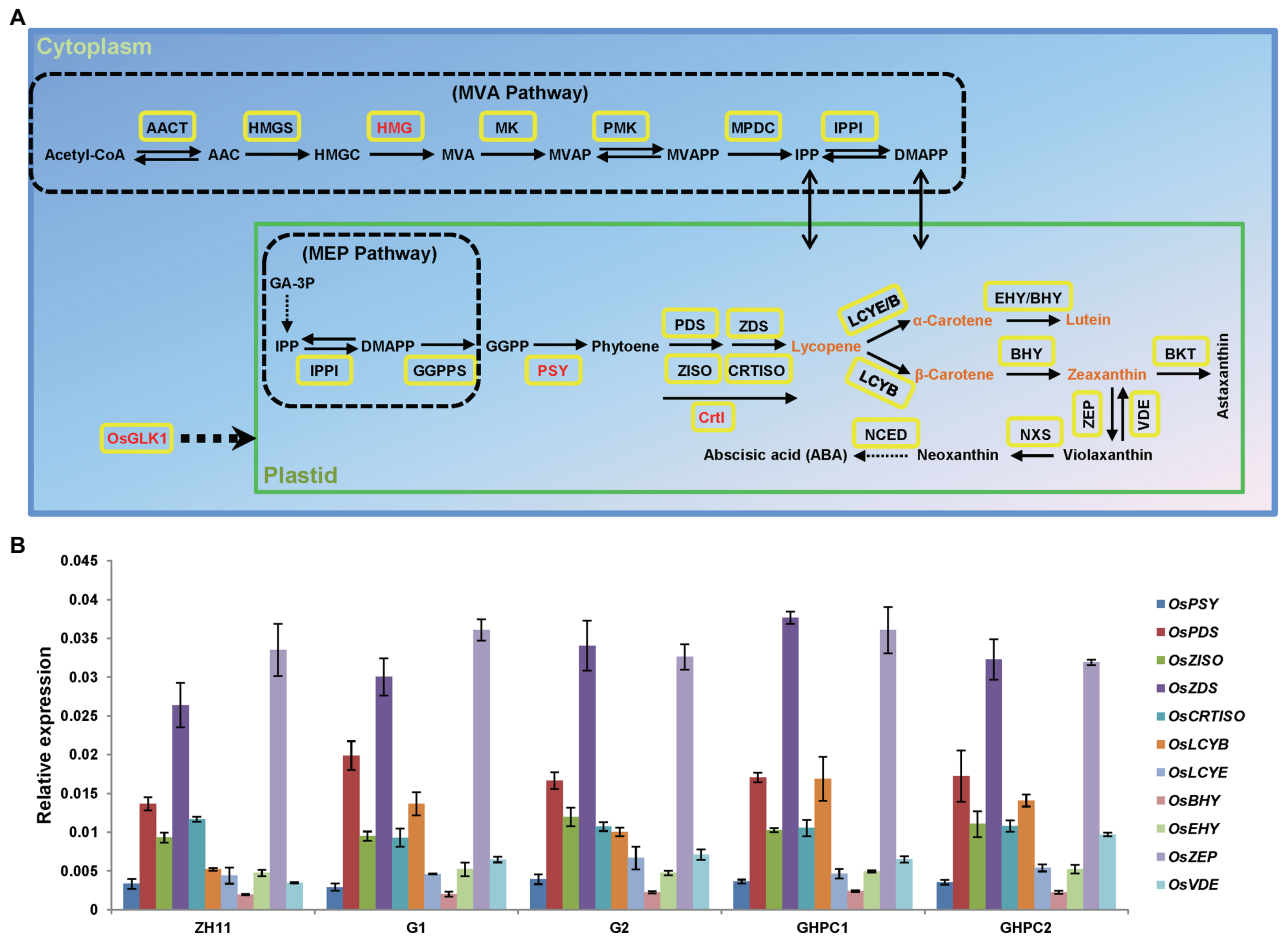
Reconstruction of simplified and expanded carotenoid biosynthesis pathway in rice endosperm (Vranová et al., 2013; Nisar et al., 2015) and qRT-PCR analysis of related endogenous carotenogenic genes in ZH11 and transgenic lines. **(A)** Carotenoid biosynthesis pathway combined with MVA pathway in plants. Various conversion steps are indicated with arrows. Yellow boxes represent the genes catalyzing the various reactions. Four transgenes were indicated with red font. AACT, acetoacetyl-CoA thiolase; AAC, acetoacetyl-CoA; HMGS, 3-hydroxy-3-methylglutaryl-CoA synthase; HMGC, 3-hydroxy-3-methylglutaryl-CoA; HMG, 3-hydroxy-3-methylglutaryl-CoA reductase; MVA; mevalonic acid; MK, MVA kinase; MVAP, mevalonate-5-phosphate; PMK, phospho-MVA kinase; MVAPP; mevalonate-5-diphosphate; MPDC, MVA diphosphate decarboxylase; GA-3P, glyceraldehydes 3-phosphate; IPP, isopentenyl diphosphate; IPPI, isopentenyl diphosphate isomerase; DMAPP, dimethylallyl diphosphate; GGPP, geranylgeranyl diphosphate; GGPPS, GGPP synthase; CrtI, bacterial (*Pantoea ananatis*) phytoene desaturase; PSY, phytoene synthase; PDS, phytoene desaturase; ZDS, ξ-carotene desaturase; ZISO, ξ-carotene isomerase; CRTISO, carotenoid isomerase; LCYB, lycopene β-cyclase; LCYE, lycopene ε-cyclase; BHY, β-ring carotene hydroxylase; EHY, ε-ring carotene hydroxylase; BKT, β-carotene ketolase; ZEP, zeaxanthin epoxidase; VDE, violaxanthin de-epoxidase; NXS, neoxanthin synthase; NCED, 9-cis epoxycarotenoid dioxygenase. **(B)** qRT-PCR analysis of endogenous carotenoid biosynthetic genes. RNA samples were prepared from endosperms of developing seeds (9 days after pollination) of the homologous T3 lines. The expression levels of the target genes were normalized to the expression of reference gene *OsActin*. Values represent means of three replicates ± SD.

In accordance with the previous literatures (Hirschberg, 2001; Vranová et al., 2013; Giuliano, 2014; Nisar et al., 2015), we summarized the expanded carotenoid biosynthetic pathway in plants (Figure 3A). In order to examine how differences in the carotenoid complement may be linked to differential changes in endogenous gene expression, qRT-PCR was used to investigate the accumulation of transcripts corresponding to the 11 genes of the carotenoid biosynthetic pathway in transgenic rice. Two lines of G and GHPC genotypes, respectively, were selected for the gene expression analysis. All of the analyzed endogenous carotenogenic genes were found to be expressed, some at relatively low levels, in the endosperm of ZH11 seeds. The mRNA levels of most endogenous genes were not greatly interfered by the overexpression of transgenes in both G and GHPC lines (Figure 3B). It is noteworthy that the transcript levels of some endogenous genes (*OsPDS*, *OsZDS*, *OsLCYB*, and *OsVDE*) were slightly up-regulated in the transgenic lines, probably due to positive feedback regulation in response to the overexpression of transgenes, which is common in the regulation of cellular processes (Schaub et al., 2005; Mitrophanov and Groisman, 2008) and also was observed in some carotenogenic transgenic plants (Huang et al., 2013; Bai et al., 2016). The enhanced expression level of endogenous carotenogenic gene *OsLCYB* might play a part in the higher accumulation of β-carotene in G and GHPC endosperms. In another branch, the expression level of *OsLCYE* was relatively low in all genotypes, but obviously sufficient for the enzyme activity to catalyze lycopene into α-carotene (Figure 3B).

### Increased grain yield and deteriorated grain quality

**Figure 4:**
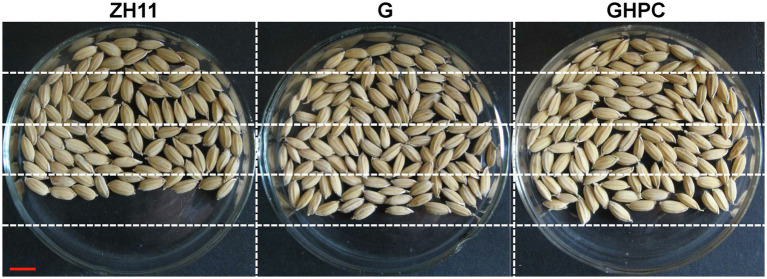
Comparison of filled grain number per panicle between ZH11, G and GHPC genotypes (Bar = 5 mm).

Rice is one of the most important food crops in the world. In order to provide a comprehensive assessment for the application of our transgenic plants, important developmental and economic traits, such as grain yield and quality, were investigated in our study. A phenotypic evaluation of G, GHPC and nontransgenic ZH11 plants revealed no major differences at the vegetative growth stage. To evaluate yield potential of G and GHPC rice lines in field conditions, we performed experiments at two different locations (Beijing and Hainan) that have distinctive climates and day lengths. Hainan is warmer than Shanghai throughout most of the season. Table 2 shows that, at both field sites, plant vegetative traits including height, tiller and panicle number, panicle length, and grain number of per panicle were not significantly changed in both G and GHPC lines compared to those in ZH11, whereas the seed setting rates of G and GHPC lines were consistently greater than those of ZH11 with differences being significant at both sites. Meanwhile, the 1,000-grain weight of G and GHPC genotypes was both slightly decreased compared with that of ZH11 (Table 2). Despite the reduced 1,000-grain weight, higher seed-setting percentage, an ~12% increase in seed-setting rate in G and GHPC lines, translated into a 17.6%–23.9% increase in grain yield (Table 2; Figure 4).

**Table 2:** Grain yield and yield components under normal field conditions in Shanghai and Hainan.

**Lines**	**Shanghai**								
	**Height (cm)**	**No. of tillers per plant**	**Panicle number per plant**	**Panicle length (cm)**	**No. of grains per panicle**	**Filled grains per panicle**	**Seed-setting rate (%)**	**1,000-grain weight (g)**	**Grain yield per plant (g)**
ZH11	81.7 ± 2.06	11.50 ± 1.90	10.50 ± 1.58	18.79 ± 1.19	113.80 ± 16.33	93.22 ± 14.70	81.95 ± 5.93	21.08 ± 0.43	19.89 ± 2.99
G1	82.3 ± 0.82	11.83 ± 1.94	11.17 ± 1.60	18.86 ± 0.53	121.42 ± 13.18	113.09 ± 13.09^*^	93.08 ± 1.34^***^	20.45 ± 0.26^**^	24.26 ± 4.07^*^ (21.97%)
G2	81.4 ± 1.51	11.57 ± 1.71	10.71 ± 2.28	18.80 ± 0.59	123.87 ± 7.53	117.11 ± 6.93^**^	94.56 ± 1.46^***^	20.53 ± 0.16^**^	24.37 ± 5.32^*^ (22.52%)
G3	81.5 ± 3.15	11.83 ± 2.23	11.17 ± 2.53	18.47 ± 0.39	118.38 ± 6.84	109.78 ± 5.55^*^	92.83 ± 3.88^**^	20.60 ± 0.17^*^	23.77 ± 6.90^*^ (19.51%)
GHPC1	81.8 ± 4.36	11.67 ± 1.37	10.83 ± 1.47	19.41 ± 0.62	124.84 ± 5.79	114.97 ± 4.87^**^	92.14 ± 2.61^**^	20.55 ± 0.14^*^	23.90 ± 2.76^*^ (20.16%)
GHPC2	81.1 ± 2.97	11.43 ± 1.62	10.57 ± 1.62	19.18 ± 0.58	120.43 ± 5.96	112.45 ± 7.38^**^	93.34 ± 2.51^***^	20.43 ± 0.17^**^	23.39 ± 4.36^*^ (17.6%)
GHPC3	81.8 ± 2.40	12.17 ± 1.33	10.67 ± 1.03	18.95 ± 0.83	121.90 ± 2.38	113.68 ± 2.80^**^	93.27 ± 1.98^***^	20.62 ± 0.15^*^	23.94 ± 2.15^*^ (20.36%)
**Lines**	**Hainan**								
	**Height (cm)**	**No. of Tillers per plant**	**Panicle number per plant**	**Panicle length (cm)**	**No. of grains per panicle**	**Filled grains per panicle**	**Seed-setting rate (%)**	**1,000-grain weight (g)**	**Grain yield per plant (g)**
ZH11	82.1 ± 2.33	11.70 ± 1.70	10.70 ± 1.89	19.10 ± 0.88	118.56 ± 13.57	97.69 ± 11.42	82.48 ± 4.81	21.21 ± 0.38	20.01 ± 2.58
G1	82.8 ± 2.14	12.17 ± 1.17	11.67 ± 1.37	19.25 ± 0.45	125.05 ± 6.96	113.05 ± 5.93^**^	90.49 ± 3.04^**^	20.57 ± 0.08^**^	23.96 ± 2.52^*^ (19.74%)
G2	82.4 ± 2.7	11.71 ± 2.14	11.14 ± 2.11	18.97 ± 0.62	119.98 ± 7.08	114.91 ± 6.35^**^	95.80 ± 1.51^***^	20.69 ± 0.27^**^	24.51 ± 4.86^*^ (22.49%)
G3	82.7 ± 1.37	12.50 ± 1.87	11.33 ± 1.97	18.76 ± 0.61	122.94 ± 4.80	116.67 ± 4.26^**^	94.91 ± 1.23^***^	20.58 ± 0.17^**^	24.79 ± 4.48^*^ (23.89%)
GHPC1	83.5 ± 2.94	11.50 ± 1.05	11.17 ± 0.75	19.02 ± 0.36	122.93 ± 5.27	114.76 ± 4.41^**^	93.40 ± 2.52^***^	20.73 ± 0.20^*^	24.43 ± 1.59^**^ (22.09%)
GHPC2	83.1 ± 3.02	12.14 ± 1.77	10.86 ± 1.21	19.17 ± 0.62	119.37 ± 6.29	111.51 ± 6.17^*^	93.43 ± 2.72^***^	20.61 ± 0.13^**^	23.62 ± 3.96^*^ (18.04%)
GHPC3	82.3 ± 1.75	12.33 ± 1.03	10.67 ± 1.03	19.03 ± 0.79	121.99 ± 4.28	115.43 ± 3.57^**^	94.64 ± 1.26^***^	20.82 ± 0.21^*^	24.25 ± 3.53^*^ (21.19%)

Values are mean ± SD (*n* = 30). Asterisks denote Student’s *t*-test significance compared with ZH11 control: ^*^p < 0.05, ^**^p < 0.01, and ^***^p < 0.001. The percentages in parentheses indicate percentages of grain yield increase compared with ZH11.

**Figure 5:**
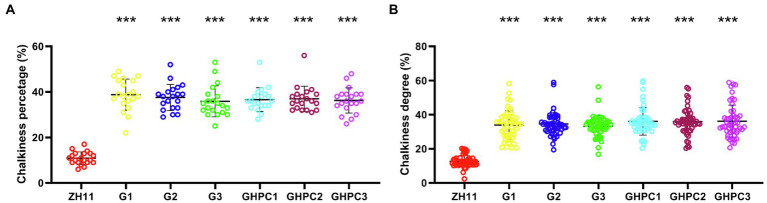
Comparison of quality traits between ZH11, G and GHPC lines. **(A)** Percentage of chalkiness (*n* = 20). **(B)** Degree of chalkiness (*n* = 50). Data are given as means ± SD. Student’s *t*-test was used to generate the *p*-values: ^***^p  < 0.001.

**Figure 6:**
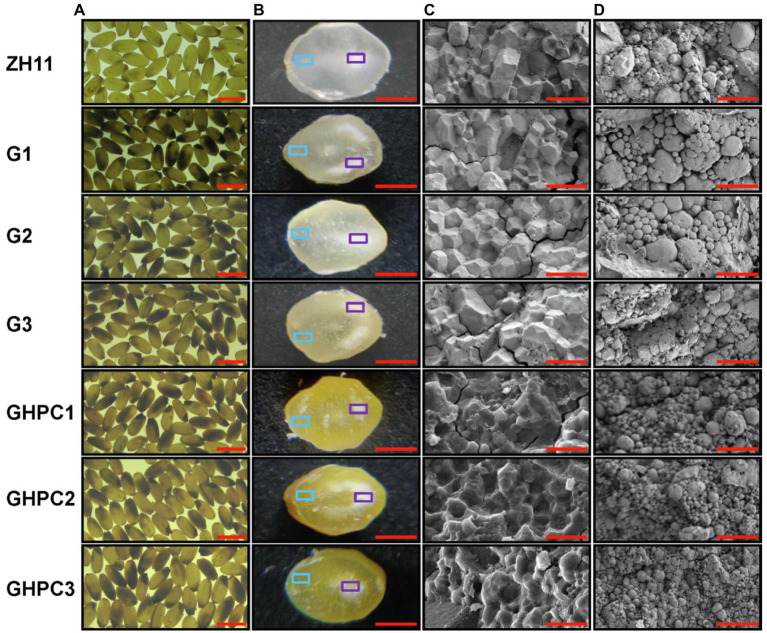
Endosperm phenotypes of rice grains of ZH11, G and GHPC plant. **(A)** Mature seeds in white light background. **(B)** Cross sections of mature endosperm. The cross-sections of the sliced grains of the transgenic lines showed that the pigments were evenly distributed in the endosperm of the grains. **(C)** SEM of the mature endosperm, with the cross sections indicated by a blue square in **(B)**. **(D)** SEM of the mature endosperm, with the cross sections indicated by a purple square in **(B)**. SEM, scanning electron microscopy. Scale bars: 5 mm **(A)**; 1 mm **(B)**; 20 μm **(C,D)**.

Surprisingly, the percentage and degree of chalkiness in G and GHPC were seriously increased compared with those in ZH11 (Figures 5A,B). The above results suggest that *OsGLK1* and/or the carotenoid overproduction might be involved in chalky endosperm formation. In the white light background, we can easily observe that the ZH11 grains displayed lower chalkiness compared with the G and GHPC grains (Figures 6A,B). The results of scanning electron microscopy revealed that the starch granules in the crystal areas of ZH11, G and GHPC grains were regular-shaped and closely arranged (Figure 6C). While, the starch granules in the chalky area of G lines were spherical, loosely packed and irregularly polyhedron-shaped (Figure 6D). The endosperms of GHPC lines consist of relatively smaller spherical starch granules (Figure 6D). In addition, waxy starch granule phenotype was observed in the endosperm of GHPC grains which might be due to the extremely high accumulation of carotenoids (Figure 6C).

## Discussion

The generation of Golden Rice 2 variety (Paine et al., 2005) justified the effectiveness of carotenoid biofortification by introducing functional genes involved in carotenoid biosynthetic pathway. For this purpose, *tHMG1*, *ZmPSY1*, and *PaCrtI* were chemically synthesized with codon optimization for rice based on the protein sequences of truncated *S. cerevisiae HMGR*, maize *PSY1*, and *P. ananatis CrtI*, respectively (Tian et al., 2019). The three gene expression cassette controlled by endosperm-specific promoter *GluB-1* from the rice globulin gene was transformed to ZH11 and greatly enhanced carotenoid production (Tian et al., 2019).

Carotenoids are synthesized in chloroplasts, chromoplasts, and other plastids in plants (Cazzonelli and Pogson, 2010; Ruiz-Sola and Rodríguez-Concepción, 2012; Sun et al., 2018). Due to the positive effects of *GLK* genes on plastid and thylakoid grana stacks development (Rossini et al., 2001; Fitter et al., 2002), it is tempting to reason that introducing a rice *GLK* transcription factor gene (*OsGLK1*), along with those for *tHMG1*, *ZmPSY1*, and *PaCrtI* may contribute to effective biofortification of carotenoids in rice endosperm. Enhancement of carotenoid production in G and GHPC lines indicated that *OsGLK1* has the potential to manipulate the plastid sink strength and enhance the availability of storage structures in rice grains. The overexpression of *OsGLK1* could be used as a “push” strategy on increasing metabolic flux in plastids to enlarge the carotenoid pool size.

Both the Golden Rice and Golden Rice 2 lines accumulated predominantly β-carotene, but phytoene, the immediate product of PSY, was completely exhausted (Ye et al., 2000; Paine et al., 2005). The buildup of β-carotene is likely to affect the resource balance in the pathway and block further conversion, especially of downstream metabolites. In other words, biosynthetic and degradative reactions and subcellular environments for deposition and sequestration within and outside of plastids have the potential to affect the final carotenoid composition. Therefore, a further enhancement of carotenoid production in rice may not only require the supply of more isoprenoid precursors by expressing functional genes of the upstream MEP and MVA pathways, but also the downstream biosynthetic genes and regulators of plastid development, such as GLKs. The data presented here revealed that the insertion of *OsGLK1* in the multigene-stacking expression cassette is an effective strategy to significantly boost carotenoid biosynthesis in rice endosperm. Furthermore, we have noticed that the total carotenoid levels in our GHPC lines are comparable to those of classic *PSY/CrtI* combination (up to 36.7 μg/g; Paine et al., 2005). The carotenoid content in *ZmPSY1/PaCrtI* combination (PC lines) reported by our lab ranged from 7.19 to 8.05 μg/g (Tian et al., 2019). The four genes used in this study were chemically synthesized and the codons were optimized in accordance with the codon usage frequencies of rice. In addition, different plant expression vectors and different transgenic recipient varieties were employed. All these factors combined might have caused a distinction in terms of carotenoid accumulation between our lines and those from Paine et al. (2005). The growing conditions could also contribute to differences in performance.

The expression analysis of endogenous carotenoid biosynthetic genes implicated weak quantitative association between steady-state transcript levels and pigment accumulation. Although most of the carotenoid biosynthetic genes were not differentially expressed in transgenic rice grains compared with controls, the higher plastid numbers and better developed thylakoid presented a source of more carotenoids, which increased the sink capacity for carotenoid accumulation (Lu and Li, 2008). Moreover, the flux of substrate into either branch of xanthophyll formation is controlled by the two *OsLCYs*, converting lycopene into α-carotene and β-carotene. The change in transcript level of *OsLCYB* might contribute to the accumulation of downstream carotenoids. In addition, more lutein than zeaxanthin was found both in G and GHPC lines, which indicated a crucial role of *BHY*. *OsLCYB* and *BHY* could serve as metabolic bottlenecks in the carotenoid biosynthesis pathway, which will help to update strategies for the generation of engineered plants with specific carotenoid profiles or higher levels of carotenoids.

Certain carotenoids, most importantly β-carotene, are cleaved to vitamin A within the body and are referred to as pro-vitamin A (Yeum and Russell, 2002). Vitamin A deficiency, a serious nutritional problem of children all over the developing world, can result in growth retardation in children and seriously impair vision and increase the incidence and severity of infectious diseases (Stephensen, 2001). In Asia, rice is predominantly consumed but lacks pro-vitamin A in the edible part of the grain (endosperm). Biofortification of carotenoids in a staple food such as rice could be of great significance. We all know that an extremely complicated mechanism coordinates carotenoid gene expression, enzyme activities, and plastid differentiation to ensure an appropriate production of carotenoids. In this study, we demonstrate that genetic engineering using *OsGLK1* driven by the endosperm-specific promoter can biofortify the carotenoid biosynthesis to a great extent in the rice endosperm. Our results made a step forward to show the critical influence of plastid identity for carotenoid accumulation. The comparison of metabolic products from GHPC plants and HPC plants from our previous report confirmed that combined expression of the four exogenous genes (*OsGLK1*, *tHMG1*, *ZmPSY1*, and *PaCrtI*) is necessary for attaining higher production of carotenoid in rice endosperm. The GHPC lines reported here has up to 40 μg/g carotenoid, of which nearly 95.7% is α- and β-carotene. This increase in total carotenoid and proportion of α- and β-carotene over the original HPC lines is of great benefit to vitamin A deficiency and related health problems. Moreover, our G and GHPC lines showed increased grain yield and seed-setting rates which were not observed in HPC lines (Tian et al., 2019). It might be safe to conclude that the endosperm-specific overexpression of *OsGLK1* plays a positive role in these two economic traits of rice.

Taken together, the protocol in this study could be used to engineer different biofortified cereals that produce grains enriched with various phytonutrients. This work also facilitates knowledge-based directed biotechnological approaches for complex metabolic engineering in synthetic biology and improvement of quality traits in plants. Overall, our transgenic rice can be used as “cell factories” for producing commercially valuable novel compounds. Our results reveal that enhancing metabolic sink strength for carotenoids in plastids, particularly by promoting plastid formation and development, could hold enormous promise for biofortification of staple crops with increased carotenoid content. Moreover, *OsGLK1* gene might have potential applications for boosting plastid-localized biosynthesis of other nutrients, such as vitamins B2, E and K1, and plastid-dependent enrichment of nutritional elements, such as selenium, in rice grains.

## Data availability statement

The original contributions presented in the study are included in the article/Supplementary Material, further inquiries can be directed to the corresponding authors.

## Author contributions

ZL performed the research and wrote the manuscript. QY, YT, BW, JX, XF, LW, WZ, YD, YW, and ZG critically reviewed and revised the manuscript. JG, RP, and HH analyzed the metabolomics data. All authors contributed to the article and approved the submitted version.

## Funding

This work was supported by Innovation Team Project of Shanghai Academy of Agricultural Sciences [2022–005] and Shanghai Academic Technology Research Leader (19XD1432300). The funders had no role in study design, data collection and analysis, decision to publish, or preparation of the manuscript.

## Conflict of interest

The authors declare that the research was conducted in the absence of any commercial or financial relationships that could be construed as a potential conflict of interest.

## Publisher’s note

All claims expressed in this article are solely those of the authors and do not necessarily represent those of their affiliated organizations, or those of the publisher, the editors and the reviewers. Any product that may be evaluated in this article, or claim that may be made by its manufacturer, is not guaranteed or endorsed by the publisher.

## Footnotes

^1^ https://rapdb.dna.affrc.go.jp/

## Supplementary material

Supplementary Figure S1 HPLC analysis of carotenoids in mature rice endosperms from ZH11, G and GHPC lines. Labeled peaks: A, astaxanthin; 1, lutein; 2, zeaxanthin; 3, lycopene, 4, α-carotene; 5, β-carotene. AU, absorbance unit.

Supplementary Figure S2 Hierarchical cluster analysis (HCA) of 89 differentially produced metabolites in mature ZH11 and G endosperms. Metabolite content is presented as median-centered averages with three biological replicates each. Red and blue colors indicate high and low content, respectively.

Supplementary Figure S3 Expression levels of the three transgenes in endosperms of two HPC lines. RNA samples were prepared from endosperms of developing seeds (9 days after pollination) of the homologous T3 lines. The transcripts were normalized to the expression of reference gene *OsActin*. Values represent means of three replicates ± SD.

Supplementary Figure S4 Transmission electron micrographs of rice endosperm. **(A)** The endosperm cells (15 DAP) of HPC genotype are shown. Scale bar = 1 μm. **(B)** The endosperm cells (45 DAP) of HPC genotype are shown. Scale bar = 2 μm. PL, plastids; SG, starch grain; OD, oil droplet; CW, cell walls.

Supplementary Figure S5 Transmission electron micrographs of rice seed coats. **(A)** Phenotypes of rice seed coats from ZH11, G, and GHPC lines. Scale bar = 1 mm. **(B)** The cells of rice seed coats are shown. Scale bar = 5 μm. **(C)** Chloroplasts boxed in (B) are shown by higher magnification. Scale bar = 1 μm. The seed coats were sampled from 15 DAP rice grains of indicated genotypes.

## Abbreviations

DW: Dry weight
HPLC: High-Performance Liquid Chromatography
LC/MS: Liquid chromatography-mass spectrometry
MEP: Methylerythritol 4-phosphate pathway
MVA: Mevalonic acid pathway
PTDS: PCR-based two-step DNA synthesis
SD: Standard deviation
SEM: Scanning electron microscopy
TEM: Transmission electron microscopy
UHPLC-QTOFMS: Ultra-High-Performance Liquid Tandem Chromatography Quadrupole Time of Flight Mass Spectrometry
