# Supplementary figures and images for: Rice carotenoid biofortification and yield improvement conferred by endosperm-specific overexpression of OsGLK1
Source: Front Plant Sci. 2022 Jul 15;13:951605. doi: 10.3389/fpls.2022.951605 (PMC9335051; doi:10.3389/fpls.2022.951605)

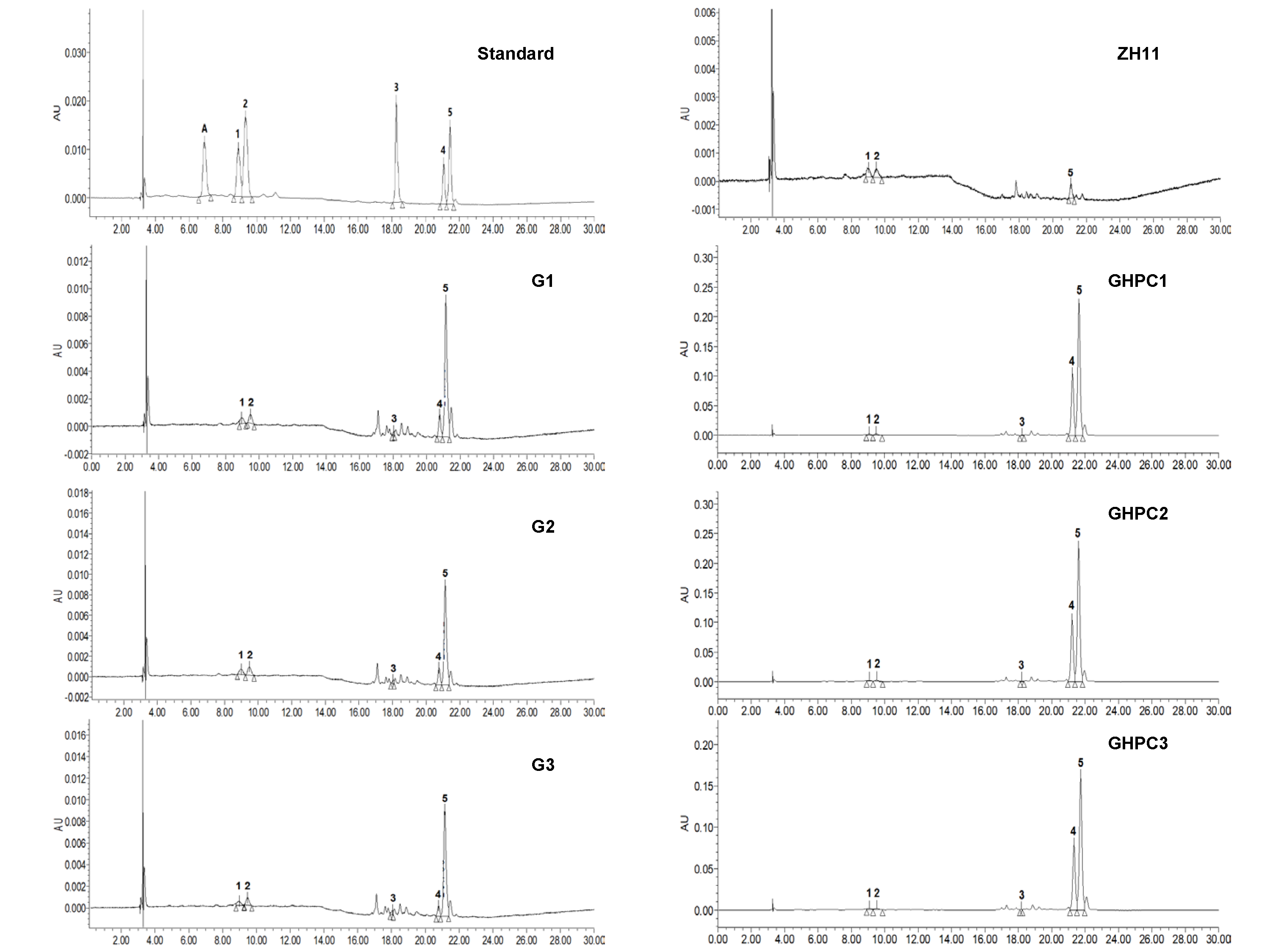

Supplement: Supplementary Figure S1 — HPLC analysis of carotenoids in mature rice endosperms from ZH11, G and GHPC lines. Labeled peaks: A, astaxanthin; 1, lutein; 2, zeaxanthin; 3, lycopene, 4, α-carotene; 5, β-carotene. AU, absorbance unit. [file Image_1.TIFF]

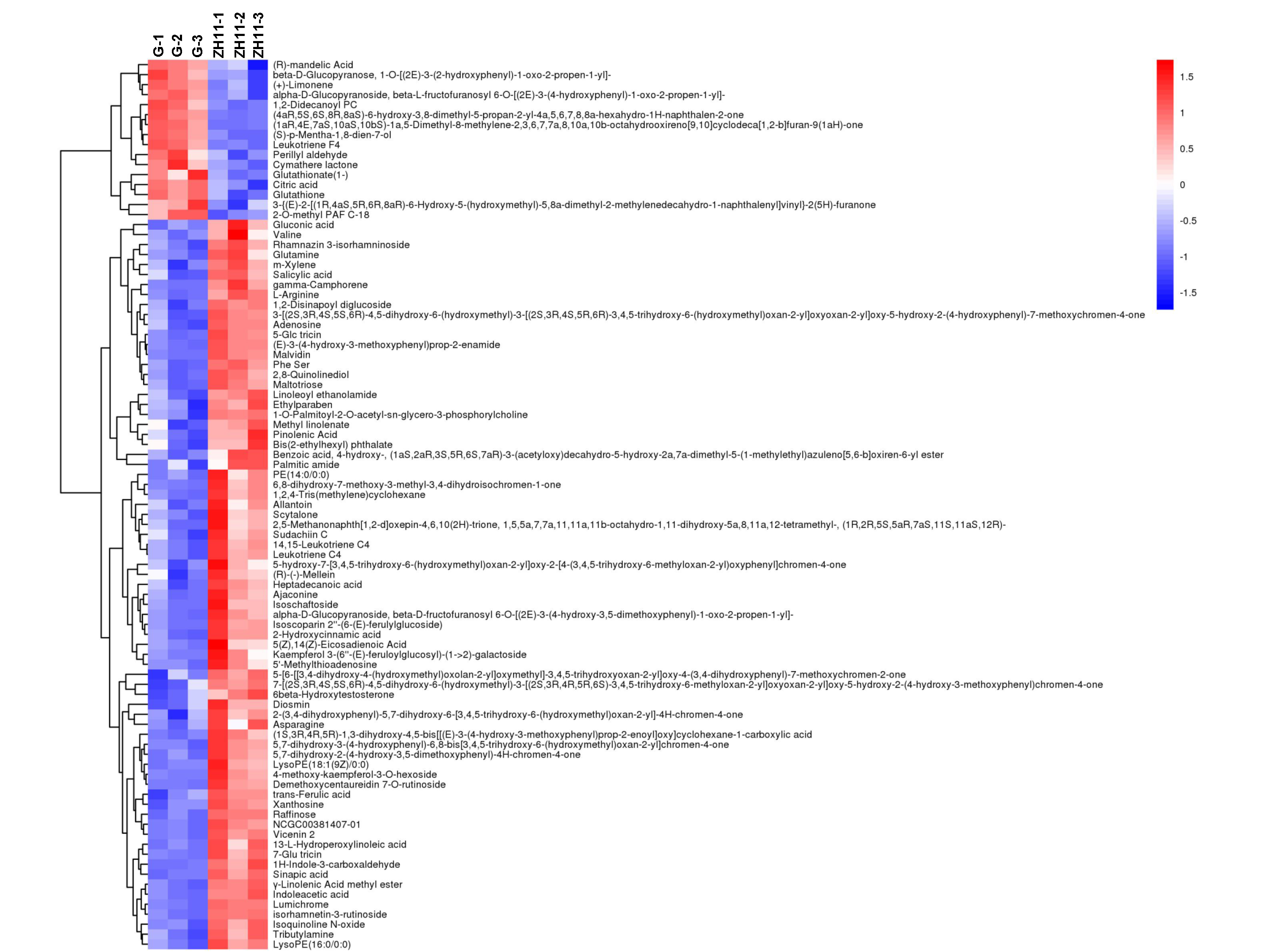

Supplement: Supplementary Figure S2 — Hierarchical cluster analysis (HCA) of 89 differentially produced metabolites in mature ZH11 and G endosperms. Metabolite content is presented as median-centered averages with three biological replicates each. Red and blue colors indicate high and low content, respectively. [file Image_2.TIFF]

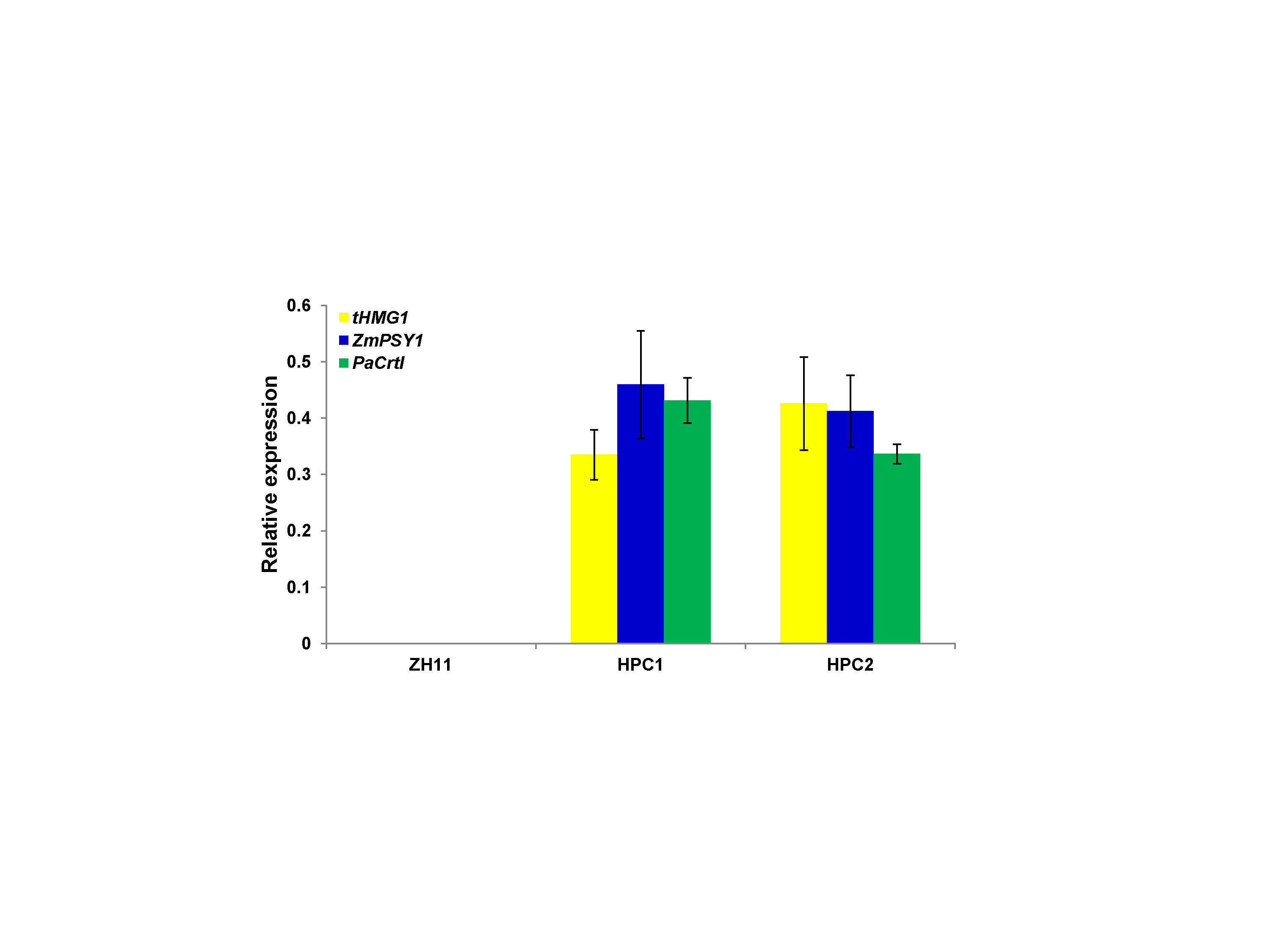

Supplement: Supplementary Figure S3 — Expression levels of the three transgenes in endosperms of two HPC lines. RNA samples were prepared from endosperms of developing seeds (9 days after pollination) of the homologous T3 lines. The transcripts were normalized to the expression of reference gene OsActin. Values represent means of three replicates ± SD. [file Image_3.TIFF]

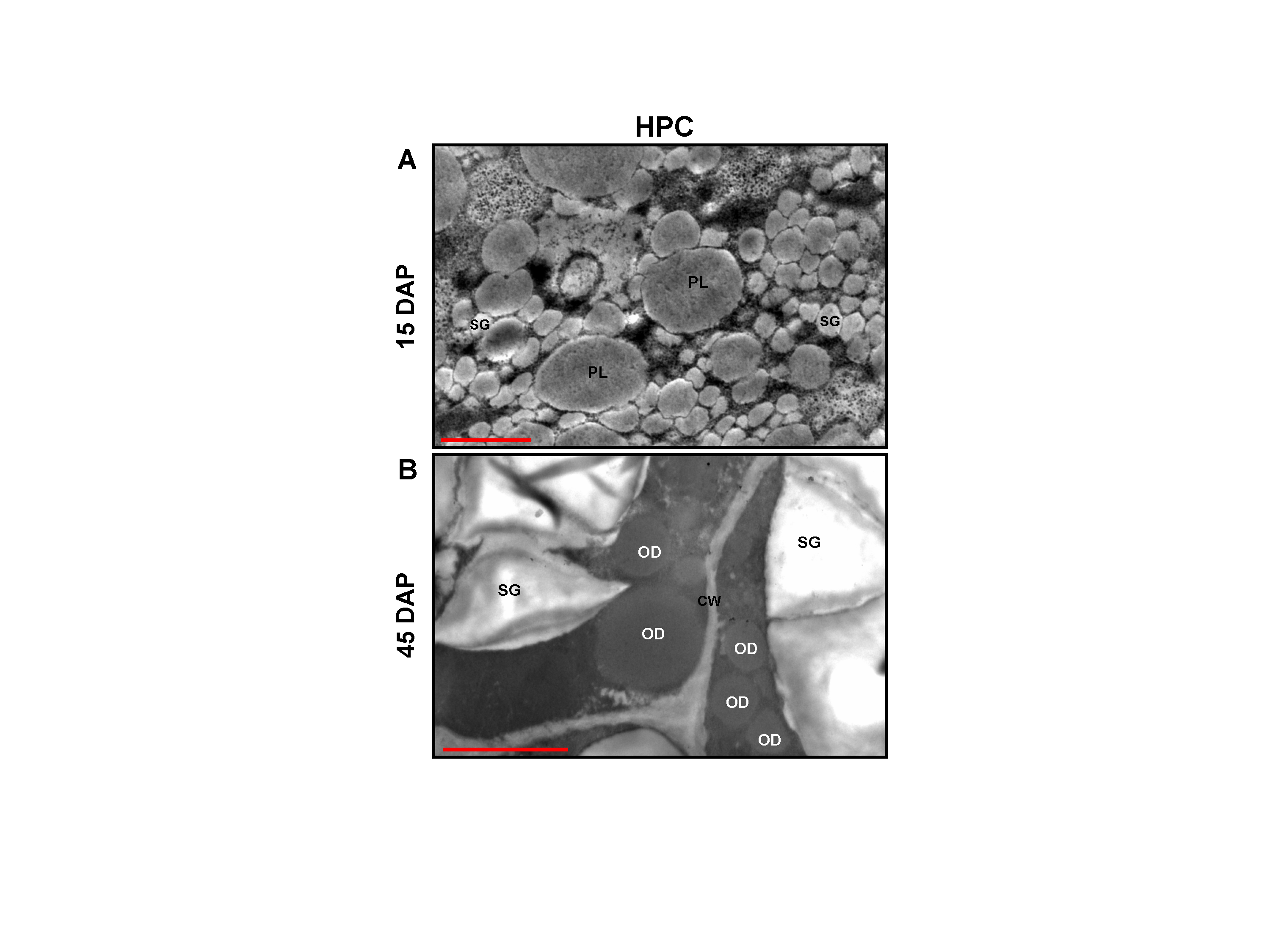

Supplement: Supplementary Figure S4 — Transmission electron micrographs of rice endosperm. (A) The endosperm cells (15 DAP) of HPC genotype are shown. Scale bar = 1 μm. (B) The endosperm cells (45 DAP) of HPC genotype are shown. Scale bar = 2 μm. PL, plastids; SG, starch grain; OD, oil droplet; CW, cell walls. [file Image_4.TIFF]

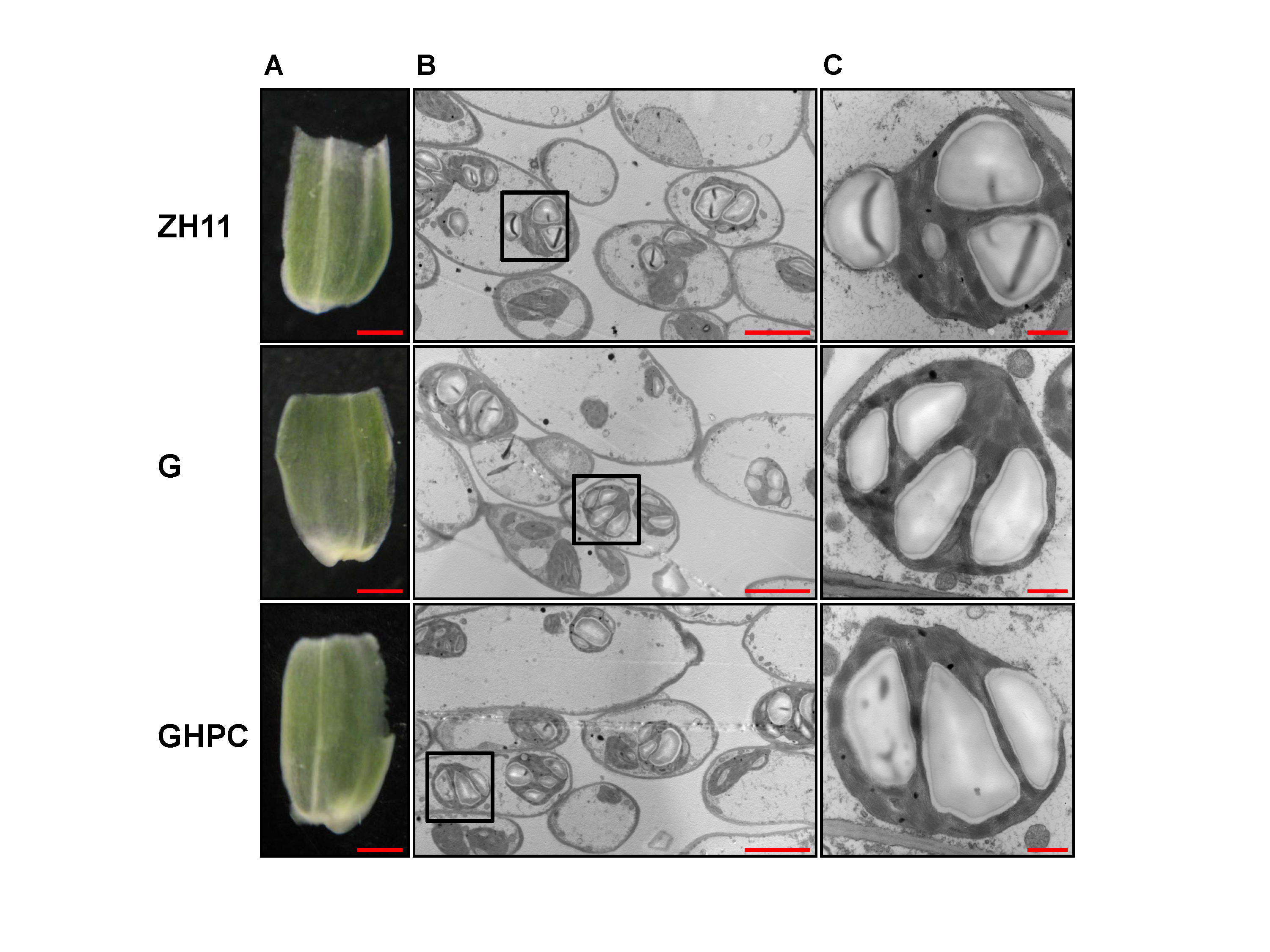

Supplement: Supplementary Figure S5 — Transmission electron micrographs of rice seed coats. (A) Phenotypes of rice seed coats from ZH11, G, and GHPC lines. Scale bar = 1 mm. (B) The cells of rice seed coats are shown. Scale bar = 5 μm. (C) Chloroplasts boxed in (B) are shown by higher magnification. Scale bar = 1 μm. The seed coats were sampled from 15 DAP rice grains of indicated genotypes. [file Image_5.TIFF]
