# Supplementary material for: Rice carotenoid biofortification and yield improvement conferred by endosperm-specific overexpression of OsGLK1
Source: Front Plant Sci. 2022 Jul 15;13:951605. doi: 10.3389/fpls.2022.951605 (PMC9335051; doi:10.3389/fpls.2022.951605)
Supplement: Supplementary file 6 [file Table_1.DOCX]

**Table S1 List of the primers used for chemical synthesis of *OsGLK1*, *tHMG1*, *ZmPSY1* and *PaCrtI*.**

| **Gene name** | **Primer sequence (5’ to 3’)** |
| --- | --- |
| *OsGLK1-1* | GGA,TCC,ATG,CTT,GCT,GTT,TCT,CCT,GCT,ATG,TGT,CCT,GAC,ATC,GAG,GAC,AGA,GCT,GCT,GTT |
| *OsGLK1-2* | CAG,AAG,ACA,TAC,CAA,CAA,CCT,CCA,TAC,CAG,CAT,CAC,CAG,CAA,CAG,CAG,CTC,TGT,CCT,CGA |
| *OsGLK1-3* | GGT,TGT,TGG,TAT,GTC,TTC,TGA,CGA,TAT,GGA,CCA,GTT,CGA,CTT,CTC,TGT,TGA,TGA,CAT,CGA |
| *OsGLK1-4* | AAC,GTC,ACC,ATC,CTC,AAG,TCT,GAG,GAA,GAA,GTC,ACC,GAA,GTC,GAT,GTC,ATC,AAC,AGA,GAA |
| *OsGLK1-5* | GAC,TTG,AGG,ATG,GTG,ACG,TTC,TTC,CTG,ATC,TTG,AGG,TTG,ATC,CTG,CTG,AAA,TCT,TCA,CTG |
| *OsGLK1-6* | TGA,ACA,CCT,TCA,CCA,CCA,GAG,GTA,GCG,ATA,GCC,TCG,AAG,TCA,GTG,AAG,ATT,TCA,GCA,GGA |
| *OsGLK1s-7* | TCT,GGT,GGT,GAA,GGT,GTT,CAG,GAT,CAG,GAG,GTT,CCA,ACT,GTT,GAA,CTC,TTG,GCT,CCT,GCT |
| *OsGLK1-8* | CAA,CAA,CGT,CAC,CAC,ATG,GAT,CGA,GAA,CAC,CAA,CAT,CAT,CAG,CAG,GAG,CCA,AGA,GTT,CAA |
| *OsGLK1-9* | TCC,ATG,TGG,TGA,CGT,TGT,TGT,TGG,TAA,GGA,GAA,TGC,TGC,TTT,CGC,TGG,TGC,TGG,AGA,GGA |
| *OsGLK1-10* | AGC,TTC,ACC,AGC,GTC,GTC,ATC,CTG,GTT,GCA,ACC,ACC,CTT,CTC,CTC,TCC,AGC,ACC,AGC,GAA |
| *OsGLK1-11* | ATG,ACG,ACG,CTG,GTG,AAG,CTA,ACG,CTG,ATG,ACG,GTG,CTG,CTG,CTG,TTG,AGG,CTA,AGT,CCT |
| *OsGLK1-12* | TCA,GCC,TCC,TGG,GAA,GAA,GAG,GTG,GAG,GAG,GAT,GGA,GAA,GAG,GAC,TTA,GCC,TCA,ACA,GCA |
| *OsGLK1-13* | TCT,TCT,TCC,CAG,GAG,GCT,GAG,TCC,AGA,CAC,AAG,TCC,TCC,TCC,AAG,TCC,TCT,CAC,GGT,AAG |
| *OsGLK1-14* | TTC,TGT,GGA,GTT,CAG,GAG,TCC,AGT,CAA,CCT,TAG,CCT,TCT,TCT,TAC,CGT,GAG,AGG,ACT,TGG |
| *OsGLK1-15* | GAC,TCC,TGA,ACT,CCA,CAG,AAG,ATT,CGT,TCA,GGC,TGT,TGA,ACA,GCT,TGG,TAT,CGA,CAA,GGC |
| *OsGLK1-16* | GGA,GTC,GAT,ACC,CAT,GAT,CTC,AAG,GAT,TCT,GGA,TGG,AAC,AGC,CTT,GTC,GAT,ACC,AAG,CTG |
| *OsGLK1-17* | AGA,TCA,TGG,GTA,TCG,ACT,CCC,TCA,CCA,GAC,ACA,ACA,TTG,CTT,CTC,ACC,TCC,AGA,AGT,ACA |
| *OsGLK1-18* | GCC,TCA,GCC,TCT,CTA,GCG,ATC,ATG,TGC,TTT,CTG,TGG,GAT,CTG,TAC,TTC,TGG,AGG,TGA,GAA |
| *OsGLK1-19* | ATC,GCT,AGA,GAG,GCT,GAG,GCT,GCT,TCT,TGG,ACT,CAG,AGA,AGA,CAA,ATC,TAT,GCT,GCT,GGT |
| *OsGLK1-20* | TCC,AAG,CGT,TGG,ACT,CAG,GTC,TCT,TAG,CAA,CAG,CAC,CAC,CAC,CAG,CAG,CAT,AGA,TTT,GTC |
| *OsGLK1-21* | ACC,TGA,GTC,CAA,CGC,TTG,GAC,TGT,TCC,AAC,CAT,CGG,TTT,CCC,ACC,ACC,ACC,ACC,ACC,ACC |
| *OsGLK1-22* | GAG,AGG,TCT,AGC,GAA,GTG,TTG,CAT,AGG,AGC,AGG,AGA,TGG,TGG,TGG,TGG,TGG,TGG,TGG,TGG |
| *OsGLK1-23* | AAC,ACT,TCG,CTA,GAC,CTC,TCC,ATG,TTT,GGG,GAC,ACC,CTA,CTA,TGG,ACC,CAT,CCA,GAG,TTC |
| *OsGLK1-24* | GCA,GGA,CCT,CTA,GGA,ACG,AGG,TGT,CTA,GGT,GGC,CAA,ACT,GGA,ACT,CTG,GAT,GGG,TCC,ATA |
| *OsGLK1-25* | CTC,GTT,CCT,AGA,GGT,CCT,GCT,CCA,CCT,TGG,GTT,CCA,CCA,CCA,CCA,CCA,TCT,GAC,CCT,GCA |
| *OsGLK1-26* | GAA,CGT,GTG,CTG,GTC,CTC,TCA,TGT,ATG,GGT,GGT,GCC,AGA,ATG,CAG,GGT,CAG,ATG,GTG,GTG |
| *OsGLK1-27* | GAG,AGG,ACC,AGC,ACA,CGT,TCC,AAC,TCA,AGG,TAC,TCC,ATG,CAT,GGC,TAT,GCC,TAT,GCC,AGC |
| *OsGLK1-28* | TGG,AAC,AAC,TCC,TGG,AAC,AGG,TGG,AGC,AGG,GAA,TCT,AGC,AGC,TGG,CAT,AGG,CAT,AGC,CAT |
| *OsGLK1-29* | CTG,TTC,CAG,GAG,TTG,TTC,CAT,GTC,CAA,TGT,ACA,GAC,CAC,TCA,CTC,CAC,CAG,CAC,TCA,CCT |
| *OsGLK1-30* | TGA,ACC,TGG,AGT,TGG,AGC,TGT,GCG,TCC,TGC,TGG,TTC,TTG,GAG,GTG,AGT,GCT,GGT,GGA,GTG |
| *OsGLK1-31* | CAG,CTC,CAA,CTC,CAG,GTT,CAG,CCA,TCC,TCT,GAG,TCC,ATC,GAT,GCT,GCT,ATC,GGT,GAT,GTC |
| *OsGLK1-32* | GCT,TGA,GTC,CGA,GTG,GGA,GTG,GCA,ACC,AAG,GCT,TGG,ACA,AGA,CAT,CAC,CGA,TAG,CAG,CAT |
| *OsGLK1-33* | ACT,CCC,ACT,CGG,ACT,CAA,GCC,ACC,ATC,CGT,TGA,CTC,TGT,CAT,GGG,TGA,GCT,TCA,GAG,ACA |
| *OsGLK1-34* | GAG,CTC,TTA,ACC,ACA,AGC,TGG,TGG,AAC,GTT,TGC,AAC,ACC,TTG,TCT,CTG,AAG,CTC,ACC,CAT |
| *tHMG1-1* | GGA,TCC,ATG,CTG,ACC,AAC,AAG,ACC,GTC,ATC,TCT,GGT,TCC,AAG,GTC,AAG,TCC,TTG,TCC,TCT |
| *tHMG1-2* | CCT,CAG,AAG,AGG,AGG,ATG,GAC,CAG,AAG,AGG,AGG,ACT,GTG,CAG,AGG,ACA,AGG,ACT,TGA,CCT |
| *tHMG1-3* | TCC,ATC,CTC,CTC,TTC,TGA,GGA,AGA,CGA,CTC,TCG,TGA,TAT,TGA,GTC,CTT,GGA,CAA,GAA,GAT |
| *tHMG1-4* | ACC,AGA,GGA,CAA,CAA,GGC,TTC,CAG,TTC,TTC,GAG,TGG,ACG,GAT,CTT,CTT,GTC,CAA,GGA,CTC |
| *tHMG1-5* | AAG,CCT,TGT,TGT,CCT,CTG,GTA,ACA,CCA,AGC,AAC,TGA,AGA,ACA,AGG,AGG,TCG,CTG,CAT,TGG |
| *tHMG1-6* | TTC,TTC,TCC,AGT,GCG,TAC,AGT,GGC,AAC,TTA,CCG,TGG,ATG,ACC,AAT,GCA,GCG,ACC,TCC,TTG |
| *tHMG1-7* | CTG,TAC,GCA,CTG,GAG,AAG,AAG,TTG,GGT,GAC,ACT,ACT,CGT,GCT,GTT,GCT,GTC,CGT,CGT,AAG |
| *tHMG1-8* | CAG,ATG,CCA,AGA,CAG,GTG,CTT,CAG,CCA,AGA,TGG,ACA,GTG,CCT,TAC,GAC,GGA,CAG,CAA,CAG |
| *tHMG1-9* | AGC,ACC,TGT,CTT,GGC,ATC,TGA,TCG,TTT,GCC,ATA,CAA,GAA,CTA,CGA,CTA,CGA,CCG,TGT,CTT |
| *tHMG1-10* | CAG,TGG,CAT,GTA,ACC,GAT,GAC,GTT,CTC,ACA,ACA,GGC,ACC,AAA,GAC,ACG,GTC,GTA,GTC,GTA |
| *tHMG1-11* | TCA,TCG,GTT,ACA,TGC,CAC,TGC,CTG,TTG,GTG,TCA,TCG,GTC,CAC,TGG,TCA,TCG,ATG,GTA,CTT |
| *tHMG1-12* | ACC,AGG,CAA,CCC,TCA,GTA,GTG,GCC,ATA,GGG,ATG,TGG,TAG,GAA,GTA,CCA,TCG,ATG,ACC,AGT |
| *tHMG1-13* | ACT,ACT,GAG,GGT,TGC,CTG,GTC,GCA,TCT,GCA,ATG,CGT,GGT,TGC,AAG,GCA,ATC,AAC,GCT,GGT |
| *tHMG1-14* | GAG,TCA,TAC,CAT,CCT,TGG,TCA,AGA,CAG,TGG,TTG,CAC,CAC,CAC,CAG,CGT,TGA,TTG,CCT,TGC |
| *tHMG1-15* | GAC,CAA,GGA,TGG,TAT,GAC,TCG,TGG,ACC,TGT,TGT,TCG,TTT,CCC,AAC,CCT,GAA,ACG,TTC,TGG |
| *tHMG1-16* | GTT,CTG,ACC,CTC,TTC,GGA,GTC,CAG,CCA,GAT,TTT,GCA,GGC,ACC,AGA,ACG,TTT,CAG,GGT,TGG |
| *tHMG1-17* | ACT,CCG,AAG,AGG,GTC,AGA,ACG,CCA,TCA,AGA,AGG,CAT,TCA,ACT,CCA,CCT,CTC,GTT,TCG,CAC |
| *tHMG1-18* | AGC,AAG,TCA,CCA,GCG,AGA,CAG,GTC,TGA,ATG,TGT,TGC,AGA,CGT,GCG,AAA,CGA,GAG,GTG,GAG |
| *tHMG1-19* | TGT,CTC,GCT,GGT,GAC,TTG,CTC,TTC,ATG,CGT,TTT,CGT,ACC,ACT,ACT,GGT,GAT,GCA,ATG,GGT |
| *tHMG1-20* | GCT,TCA,AGG,AGT,ACT,CGA,CAC,CCT,TAG,AGA,TCA,TGT,TCA,TAC,CCA,TTG,CAT,CAC,CAG,TAG |
| *tHMG1-21* | TGT,CGA,GTA,CTC,CTT,GAA,GCA,GAT,GGT,CGA,AGA,GTA,TGG,TTG,GGA,AGA,CAT,GGA,GGT,TGT |
| *tHMG1-22* | AGC,AGG,CTT,CTT,GTC,GGT,GCA,GTA,GTT,ACC,AGA,AAC,GGA,GAC,AAC,CTC,CAT,GTC,TTC,CCA |
| *tHMG1-23* | GCA,CCG,ACA,AGA,AGC,CTG,CTG,CCA,TCA,ACT,GGA,TCG,AAG,GTC,GTG,GTA,AGT,CTG,TCG,TCG |
| *tHMG1-24* | AAG,ACC,TTA,CGA,ACA,ACA,TCA,CCA,GGA,ATG,GTG,GCT,TCA,GCG,ACG,ACA,GAC,TTA,CCA,CGA |
| *tHMG1-25* | GAT,GTT,GTT,CGT,AAG,GTC,TTG,AAG,TCT,GAC,GTC,TCT,GCA,CTG,GTT,GAA,CTG,AAC,ATT,GCC |
| *tHMG1-26* | CAC,CAA,CAG,AAC,CAG,CCA,TTG,CAG,AAC,CAA,CCA,GGT,TCT,TGG,CAA,TGT,TCA,GTT,CAA,CCA |
| *tHMG1-27* | AAT,GGC,TGG,TTC,TGT,TGG,TGG,TTT,CAA,CGC,ACA,TGC,TGC,CAA,CTT,GGT,CAC,TGC,TGT,CTT |
| *tHMG1-28* | AGA,CTC,GAC,GTT,CTG,TGC,AGG,ATC,TTG,ACC,CAG,TGC,CAA,GAA,GAC,AGC,AGT,GAC,CAA,GTT |
| *tHMG1-29* | CTG,CAC,AGA,ACG,TCG,AGT,CTT,CCA,ACT,GCA,TCA,CCC,TGA,TGA,AGG,AAG,TTG,ACG,GTG,ACT |
| *tHMG1-30* | GTA,CCA,ACT,TCG,ATG,GAT,GGC,ATG,GAG,ACA,GAG,ATA,CGC,AAG,TCA,CCG,TCA,ACT,TCC,TTC |
| *tHMG1-31* | CCA,TCC,ATC,GAA,GTT,GGT,ACT,ATT,GGT,GGT,GGT,ACT,GTT,CTG,GAA,CCA,CAA,GGT,GCA,ATG |
| *tHMG1-32* | GAG,CAG,TTG,CAT,GTG,GAC,CAC,GAA,CAC,CCA,ACA,AGT,CCA,GCA,TTG,CAC,CTT,GTG,GTT,CCA |
| *tHMG1-33* | TGG,TCC,ACA,TGC,AAC,TGC,TCC,TGG,TAC,TAA,CGC,ACG,TCA,ACT,GGC,ACG,TAT,CGT,TGC,ATG |
| *tHMG1-34* | CAG,TGC,AGC,ACA,CAA,GGA,CAA,CTC,ACC,AGC,CAG,GAC,AGC,ACA,TGC,AAC,GAT,ACG,TGC,CAG |
| *tHMG1-35* | TGT,CCT,TGT,GTG,CTG,CAC,TGG,CTG,CTG,GTC,ATC,TGG,TTC,AAT,CCC,ACA,TGA,CTC,ACA,ATC |
| *tHMG1-36* | GCA,TCC,AGG,TTG,TTA,GGT,TTG,GTT,GGT,TCA,GCA,GGT,TTA,CGA,TTG,TGA,GTC,ATG,TGG,GAT |
| *tHMG1-37* | AAA,CCT,AAC,AAC,CTG,GAT,GCC,ACT,GAC,ATC,AAC,CGT,CTG,AAG,GAT,GGT,TCC,GTC,ACC,TGC |
| *tHMG1-38* | GAG,CTC,TTA,GGA,TTT,GAT,GCA,GGT,GAC,GGA,ACC,ATC,CT |
| *ZmPSY1-1* | GGA,TCC,ATG,GCC,ATC,ATA,CTC,GTT,AGG,GCT,GCC,TCT,CCT,GGT,CTC,TCT,GCT,GCT,GAC,AGC |
| *ZmPSY1-2* | TGA,GCA,GGG,TGG,AGC,ACT,GGA,GAG,TCC,CCT,GGT,GGC,TGA,TGC,TGT,CAG,CAG,CAG,AGA,GAC |
| *ZmPSY1-3* | CCA,GTG,CTC,CAC,CCT,GCT,CAA,GAC,GAA,GAG,GCC,TGC,TGC,ACG,TCG,GTG,GAT,GCC,TTG,CTC |
| *ZmPSY1-4* | AGA,AGG,ACG,ACC,AGC,TTC,CCA,CGG,GTG,GAG,ACC,AAG,GAG,CGA,GCA,AGG,CAT,CCA,CCG,ACG |
| *ZmPSY1-5* | GGG,AAG,CTG,GTC,GTC,CTT,CTC,CTG,CCG,TCT,ACT,CCA,GCC,TCG,CCG,TCA,ACC,CGG,CGG,GAG |
| *ZmPSY1-6* | ACG,ACG,TCG,TAG,ACC,TTC,TGC,TCG,GAC,GAG,ACG,ACG,GCC,TCT,CCC,GCC,GGG,TTG,ACG,GCG |
| *ZmPSY1-7* | CAG,AAG,GTC,TAC,GAC,GTC,GTG,CTC,AAG,CAG,GCC,GCA,TTG,CTC,AAA,CGC,CAG,CTG,CGC,ACG |
| *ZmPSY1-8* | GTG,GCA,TGT,CCA,TGT,CCT,GGG,GCC,TGG,CGT,CGA,GGA,CCG,GCG,TGC,GCA,GCT,GGC,GTT,TGA |
| *ZmPSY1-9* | CCA,GGA,CAT,GGA,CAT,GCC,ACG,CAA,CGG,GCT,CAA,GGA,AGC,CTA,CGA,CCG,CTG,CGG,CGA,GAT |
| *ZmPSY1-10* | CAT,AGT,TCC,GAG,GTA,AAA,CGT,CTT,GGC,ATA,CTC,CTC,ACA,GAT,CTC,GCC,GCA,GCG,GTC,GTA |
| *ZmPSY1-11* | CGT,TTT,ACC,TCG,GAA,CTA,TGT,TGA,TGA,CAG,AGG,AGC,GGC,GCC,GCG,CCA,TAT,GGG,CCA,TCT |
| *ZmPSY1-12* | GGC,CCA,TCT,ACA,AGC,TCA,TCT,GTC,CTC,CTA,CAC,CAC,ACA,TAG,ATG,GCC,CAT,ATG,GCG,CGG |
| *ZmPSY1-13* | GAT,GAG,CTT,GTA,GAT,GGG,CCA,AAC,GCC,AAC,TAC,ATT,ACA,CCA,ACA,GCT,TTG,GAC,CGG,TGG |
| *ZmPSY1-14* | CGT,AAG,GAC,GTC,CCG,TGA,ACA,GAT,CCT,CAA,GTC,TCT,TCT,CCC,ACC,GGT,CCA,AAG,CTG,TTG |
| *ZmPSY1-15* | GTT,CAC,GGG,ACG,TCC,TTA,CGA,CAT,GCT,TGA,TGC,CGC,TCT,CTC,TGA,TAC,CAT,CTC,AAG,GTT |
| *ZmPSY1-16* | CCC,TTC,AAT,CAT,GTC,CCT,GAA,TGG,CTG,AAT,GTC,TAT,GGG,GAA,CCT,TGA,GAT,GGT,ATC,AGA |
| *ZmPSY1-17* | TCA,GGG,ACA,TGA,TTG,AAG,GGA,TGA,GGA,GTG,ATC,TTA,GGA,AGA,CAA,GGT,ATA,ACA,ACT,TCG |
| *ZmPSY1-18* | ACA,GTT,CCA,GCA,ACA,TAG,TAG,CAG,TAC,ATG,TAG,AGT,TCG,TCG,AAG,TTG,TTA,TAC,CTT,GTC |
| *ZmPSY1-19* | TAC,TAT,GTT,GCT,GGA,ACT,GTC,GGG,TTA,ATG,AGC,GTA,CCT,GTG,ATG,GGC,ATC,GCA,ACC,GAG |
| *ZmPSY1-20* | CCA,GGG,CAG,CAC,TGT,ACA,CGC,TTT,CAG,TTG,TTG,CTT,TAG,ACT,CGG,TTG,CGA,TGC,CCA,TCA |
| *ZmPSY1-21* | CGT,GTA,CAG,TGC,TGC,CCT,GGC,TCT,GGG,AAT,TGC,GAA,CCA,ACT,CAC,GAA,CAT,ACT,CCG,GGA |
| *ZmPSY1-22* | TGG,TAA,ATA,TAT,CCT,TCC,TCT,TCT,AGC,ATC,CTC,TCC,AAC,ATC,CCG,GAG,TAT,GTT,CGT,GAG |
| *ZmPSY1-23* | GAG,GAA,GGA,TAT,ATT,TAC,CAC,AAG,ATG,AGC,TTG,CAC,AGG,CAG,GGC,TCT,CTG,ATG,AGG,ACA |
| *ZmPSY1-24* | ATG,AAG,TTT,CTC,CAC,CGG,TTC,GTG,ACG,ACC,CCT,TTG,AAG,ATG,TCC,TCA,TCA,GAG,AGC,CCT |
| *ZmPSY1-25* | AAC,CGG,TGG,AGA,AAC,TTC,ATG,AAG,AGG,CAG,ATC,AAG,AGG,GCC,AGG,ATG,TTT,TTT,GAG,GAG |
| *ZmPSY1-26* | ATC,TGC,TAG,CCT,GTG,AGA,GTT,CAG,TTA,CCC,CTC,TCT,CTG,CCT,CCT,CAA,AAA,ACA,TCC,TGG |
| *ZmPSY1-27* | ACT,CTC,ACA,GGC,TAG,CAG,ATG,GCC,AGT,ATG,GGC,TTC,CCT,GTT,GTT,GTA,CAG,GCA,GAT,CCT |
| *ZmPSY1-28* | CTT,CGT,GAA,GTT,GTT,GTA,GTC,GTT,GGC,TTC,GAT,CTC,ATC,CAG,GAT,CTG,CCT,GTA,CAA,CAA |
| *ZmPSY1-29* | ACT,ACA,ACA,ACT,TCA,CGA,AGA,GGG,CGT,ATG,TTG,GTA,AAG,GGA,AGA,AGT,TGC,TAG,CAC,TTC |
| *ZmPSY1-30* | AAT,GAA,CAT,GGG,AGC,AGT,AGC,GAT,TTT,CCA,TAT,GCC,ACA,GGA,AGT,GCT,AGC,AAC,TTC,TTC |
| *ZmPSY1-31* | GAG,CTC,TTA,GGT,CTG,GCC,ATT,TCT,CAA,TGA,ACA,TGG,GAG,CAG,TAG |
| *PaCrtI-1* | GGA,TCC,ATG,AAG,CCA,ACT,ACT,GTC,ATC,GGT,GCT,GGT,TTC,GGT,GGT,CTG,GCT,CTG,GCT,ATC |
| *PaCrtI-2* | GTT,CCA,GCA,GCA,GGA,CTG,GGA,TAC,CAG,CAG,CTT,GCA,GAC,GGA,TAG,CCA,GAG,CCA,GAC,CAC |
| *PaCrtI-3* | CCC,AGT,CCT,GCT,GCT,GGA,ACA,ACG,TGA,CAA,GCC,AGG,TGG,TCG,TGC,TTA,CGT,CTA,CGA,GGA |
| *PaCrtI-4* | AGT,GAT,GAC,AGT,TGG,ACC,AGC,GTC,GAA,AGT,GAA,ACC,CTG,GTC,CTC,GTA,GAC,GTA,AGC,ACG |
| *PaCrtI-5* | CTG,GTC,CAA,CTG,TCA,TCA,CTG,ACC,CAT,CCG,CTA,TCG,AGG,AGC,TGT,TCG,CTC,TGG,CTG,GTA |
| *PaCrtI-6* | GGA,GTG,ACT,GGC,AGC,AGC,TCG,ACG,TAC,TCC,TTC,AGC,TGC,TTA,CCA,GCC,AGA,GCG,AAC,AGC |
| *PaCrtI-7* | GAG,CTG,CTG,CCA,GTC,ACT,CCA,TTC,TAC,CGT,CTG,TGC,TGG,GAA,TCT,GGT,AAG,GTC,TTC,AAC |
| *PaCrtI-8* | GCT,GGA,TCT,GAG,CTT,CCA,GAC,GAG,TCT,GGT,CGT,TGT,CGT,AGT,TGA,AGA,CCT,TAC,CAG,ATT |
| *PaCrtI-9* | TCT,GGA,AGC,TCA,GAT,CCA,GCA,GTT,CAA,CCC,ACG,TGA,CGT,CGA,AGG,TTA,TCG,TCA,GTT,CCT |
| *PaCrtI-10* | CTT,CAG,ATA,ACC,TTC,CTT,GAA,GAC,AGC,ACG,GGA,GTA,GTC,CAG,GAA,CTG,ACG,ATA,ACC,TTC |
| *PaCrtI-11* | TCA,AGG,AAG,GTT,ATC,TGA,AGC,TCG,GTA,CTG,TCC,CAT,TCC,TGT,CCT,TCC,GTG,ACA,TGC,TGC |
| *PaCrtI-12* | GAA,CGC,CAA,GCC,TGG,AGC,TTA,GCC,AGC,TGT,GGA,GCA,GCA,CGC,AGC,ATG,TCA,CGG,AAG,GAC |
| *PaCrtI-13* | AAG,CTC,CAG,GCT,TGG,CGT,TCT,GTC,TAC,TCC,AAG,GTC,GCT,TCC,TAC,ATC,GAA,GAC,GAA,CAC |
| *PaCrtI-14* | CAC,CGA,CCA,GCA,GGG,AGT,GGA,AGG,AGA,AAG,CCT,GAC,GCA,GGT,GTT,CGT,CTT,CGA,TGT,AGG |
| *PaCrtI-15* | CCA,CTC,CCT,GCT,GGT,CGG,TGG,TAA,TCC,ATT,CGC,TAC,TTC,CTC,CAT,CTA,CAC,TCT,GAT,CCA |
| *PaCrtI-16* | ACC,ACG,TGG,GAA,CCA,GAC,ACC,CCA,TTC,ACG,TTC,CAG,AGC,GTG,GAT,CAG,AGT,GTA,GAT,GGA |
| *PaCrtI-17* | GTG,TCT,GGT,TCC,CAC,GTG,GTG,GTA,CTG,GTG,CTC,TGG,TCC,AGG,GTA,TGA,TCA,AGC,TGT,TTC |
| *PaCrtI-18* | GAG,ACA,CGA,GCG,TTC,AGG,ACG,ACT,TCA,CCA,CCC,AGG,TCC,TGA,AAC,AGC,TTG,ATC,ATA,CCC |
| *PaCrtI-19* | GTC,CTG,AAC,GCT,CGT,GTC,TCC,CAC,ATG,GAA,ACT,ACT,GGT,AAC,AAG,ATC,GAG,GCT,GTC,CAC |
| *PaCrtI-20* | AAG,CGA,CAG,CCT,GAG,TCA,GGA,AAC,GAC,GAC,CGT,CCT,CCA,GGT,GGA,CAG,CCT,CGA,TCT,TGT |
| *PaCrtI-21* | CCT,GAC,TCA,GGC,TGT,CGC,TTC,CAA,CGC,TGA,CGT,CGT,TCA,CAC,TTA,CCG,TGA,TCT,GCT,GTC |
| *PaCrtI-22* | TTG,CAG,CTT,GTT,GGA,CTG,CTT,GAC,AGC,AGC,TGG,ATG,CTG,AGA,CAG,CAG,ATC,ACG,GTA,AGT |
| *PaCrtI-23* | AGC,AGT,CCA,ACA,AGC,TGC,AAA,CTA,AGC,GTA,TGT,CCA,ACT,CTC,TGT,TCG,TCC,TGT,ACT,TCG |
| *PaCrtI-24* | ACA,GTG,TGG,TGA,GCC,AGC,TGG,TCG,TGA,TGG,TGG,TTC,AGA,CCG,AAG,TAC,AGG,ACG,AAC,AGA |
| *PaCrtI-25* | CAG,CTG,GCT,CAC,CAC,ACT,GTC,TGC,TTC,GGT,CCA,CGT,TAC,CGT,GAG,CTG,ATC,GAC,GAA,ATC |
| *PaCrtI-26* | GGT,ACA,GAG,AGA,AGT,CCT,CAG,CCA,GAC,CGT,CGT,GGT,TGA,AGA,TTT,CGT,CGA,TCA,GCT,CAC |
| *PaCrtI-27* | TGA,GGA,CTT,CTC,TCT,GTA,CCT,GCA,CGC,TCC,ATG,CGT,CAC,TGA,CTC,CTC,TCT,GGC,TCC,AGA |
| *PaCrtI-28* | GTG,TGG,GAC,TGG,AGC,CAG,AAC,GTA,GTA,GGA,ACC,ACA,ACC,TTC,TGG,AGC,CAG,AGA,GGA,GTC |
| *PaCrtI-29* | TTC,TGG,CTC,CAG,TCC,CAC,ACC,TGG,GTA,CTG,CTA,ACC,TGG,ACT,GGA,CTG,TCG,AAG,GTC,CAA |
| *PaCrtI-30* | TAG,TGC,TGT,TCC,AGG,TAA,GCG,AAG,ATA,CGG,TCA,CGC,AGC,TTT,GGA,CCT,TCG,ACA,GTC,CAG |
| *PaCrtI-31* | GCT,TAC,CTG,GAA,CAG,CAC,TAC,ATG,CCA,GGT,CTG,CGT,TCT,CAG,CTG,GTC,ACT,CAC,CGT,ATG |
| *PaCrtI-32* | GGT,AAG,CGT,TCA,GCT,GAT,CAC,GGA,AGT,CGA,ATG,GAG,TGA,ACA,TAC,GGT,GAG,TGA,CCA,GCT |
| *PaCrtI-33* | TGA,TCA,GCT,GAA,CGC,TTA,CCA,CGG,TTC,CGC,TTT,CTC,CGT,CGA,ACC,AGT,CCT,GAC,TCA,GTC |
| *PaCrtI-34* | AGT,GAT,AGT,CTT,GTC,ACG,GTT,GTG,TGG,ACG,GAA,CCA,AGC,AGA,CTG,AGT,CAG,GAC,TGG,TTC |
| *PaCrtI-35* | ACC,GTG,ACA,AGA,CTA,TCA,CTA,ACC,TGT,ACC,TGG,TCG,GTG,CTG,GTA,CTC,ATC,CAG,GTG,CTG |
| *PaCrtI-36* | CCA,GCA,GTA,GCC,TTA,GCG,GAA,CCG,ATG,ACA,CCT,GGG,ATA,CCA,GCA,CCT,GGA,TGA,GTA,CCA |
| *PaCrtI-37* | GAG,CTC,TTA,GAT,CAG,GTC,CTC,CAG,CAT,CAG,ACC,AGC,AGT,AGC,CTT,AGC,GGA |
