# Supplementary material for: Rice carotenoid biofortification and yield improvement conferred by endosperm-specific overexpression of OsGLK1
Source: Front Plant Sci. 2022 Jul 15;13:951605. doi: 10.3389/fpls.2022.951605 (PMC9335051; doi:10.3389/fpls.2022.951605)
Supplement: Supplementary file 7 [file Table_2.DOCX]

**Table S2 Primers used in real-time PCR assay.**

| Gene Name | Primer sequence (5’ to 3’) |
| --- | --- |
| *OsGLK1* | F: CTCACTCCACCAGCACTCACC |
|  | R: ACAAGCTGGTGGAACGTTTGC |
| *ZmPSY1* | F: GTAACTGAACTCTCACAGGCT |
|  | R: TCTGGCCATTTCTCAATGAAC |
| *PaCrtI* | F: TTCGACTTCCGTGATCAGCTG |
|  | R: GATCAGGTCCTCCAGCATCAG |
| *tHMG1* | F: GGTACTAACGCACGTCAACTG |
|  | R: GGATTTGATGCAGGTGACGGA |
| *OsPSY* | F: GAAGCTTCATGAAGAGACAG |
|  | R: AATCGTTTGCTTCTATCTCG |
| *OsPDS* | F: ATGGGTTGGACGGAGTGACA |
|  | R: GTTCACAGTCCGGGATAGTC |
| *OsZISO* | F: TCAGTTGCTGTTGCGGCATC |
|  | R: TGTCTTCCATCGATAACTGC |
| *OsZDS* | F: GTCATCACAAGGCTTGGAAC |
|  | R: CTTCCATGCTGTCAATGTAG |
| *OsCRTISO* | F: AATGATGGTACATATGGACC |
|  | R: ATCCCTGAAAATGCCACAGC |
| *OsLCYB* | F: ACATCCTCCTCAAGCTCGAC |
|  | R: GTTGGAGGCCTTGGCGAAGA |
| *OsLCYE* | F: GGAACCTCTCAAAGTTCCAG |
|  | R: GAATGCCTTCGTTATTCAGT |
| *OsBHY* | F: CGTACATGTTCGTCCACGAC |
|  | R: AACTCCTTGGGTCCGAGGAA |
| *OsEHY* | F: CGTACATGTTCGTCCACGAC |
|  | R: CTCCTTGGGACCAAGGAACA |
| *OsZEP* | F: TGGCATTGCCCTTGCCACAG |
|  | R: GAAACGGACAGGGAAGTTCG |
| *OsVDE* | F: AGGGAAGTGCAGGAGATCGA |
|  | R: TCTGATCCAAAAGCTCCATC |
| *OsActin* | F: TGTATGCCAGTGGTCGTACCA |
|  | R: CCAGCAAGGTCGAGACGAA |
