# Supplementary material for: Rice carotenoid biofortification and yield improvement conferred by endosperm-specific overexpression of OsGLK1
Source: Front Plant Sci. 2022 Jul 15;13:951605. doi: 10.3389/fpls.2022.951605 (PMC9335051; doi:10.3389/fpls.2022.951605)
Supplement: Supplementary file 8 [file Table_3.DOCX]

Table S3 Differentially produced metabolites in ZH11 and G1 transgenic plants (VIP > 1; *P*-value < 0.05; fold change > 2 or < 0.5)

| **Compound name** | **Polarity** | **mz** | **VIP** | ***P*-value** | **Fold change**  **(G1 vs ZH11)** |
| --- | --- | --- | --- | --- | --- |
| **Up-regulated** |  |  |  |  |  |
| (S)-p-Mentha-1,8-dien-7-ol | + | 135.116 | 1.19 | 5.2E-04 | 13.29 |
| (4aR,5S,6S,8R,8aS)-6-hydroxy-3,8-dimethyl-5-propan-2-yl-4a,5,6,7,8,8a-hexahydro-1H-naphthalen-2-one | + | 219.1751 | 1.27 | 1.8E-04 | 11.35 |
| (1aR,4E,7aS,10aS,10bS)-1a,5-Dimethyl-8-methylene-2,3,6,7,7a,8,10a,10b-octahydrooxireno[9,10]cyclodeca[1,2-b]furan-9(1aH)-one | + | 249.1468 | 1.29 | 0.006 | 11.28 |
| (+)-Limonene | + | 137.1321 | 1.20 | 0.003 | 2.05 |
| Cymathere lactone | + | 273.1836 | 1.22 | 0.007 | 2.01 |
| **Down-regulated** |  |  |  |  |  |
| Isoquinoline N-oxide | + | 146.0606 | 1.25 | 0.001 | 0.5 |
| Sinapic acid | - | 223.0616 | 1.28 | 3.1E-04 | 0.49 |
| 6,8-dihydroxy-7-methoxy-3-methyl-3,4-dihydroisochromen-1-one | + | 225.0761 | 1.25 | 0.005 | 0.49 |
| 2,8-Quinolinediol | + | 162.0546 | 1.26 | 0.001 | 0.49 |
| Maltotriose | - | 503.1633 | 1.24 | 0.002 | 0.48 |
| Lumichrome | + | 243.0872 | 1.29 | 1.9E-05 | 0.46 |
| Asparagine | + | 133.061 | 1.15 | 0.024 | 0.46 |
| Indoleacetic acid | + | 176.0706 | 1.28 | 1.5E-04 | 0.45 |
| 5(Z),14(Z)-Eicosadienoic Acid | + | 309.278 | 1.19 | 0.036 | 0.4 |
| Heptadecanoic acid | + | 271.2628 | 1.16 | 0.008 | 0.39 |
| gamma-Camphorene | + | 273.2559 | 1.27 | 0.002 | 0.35 |
| Raffinose | - | 503.1627 | 1.28 | 5.8E-05 | 0.35 |
| NCGC00381407-01 | + | 265.1488 | 1.28 | 4.5E-04 | 0.31 |

mz, ion mass-to-charge ratio; VIP, Variable Importance in the Projection
